# Gene expression analysis of *Fmr1*-KO mouse caudate putamen reveals new preclinical biomarkers for fragile X syndrome

**DOI:** 10.3389/fnins.2026.1833413

**Published:** 2026-10-23

**Authors:** Shirin Sharghi, Stefanie Flunkert, Magdalena Daurer, Livia Breznik, Boris Philippe Chagnaud, Manuela Prokesch

**Affiliations:** 1 Scantox Neuro GmbH, Grambach, Austria; 2 Institute for Biology, Karl-Franzens University of Graz, Graz, Austria; 3 BioDoks e. U., St. Georgen an der Stiefing, Austria

**Keywords:** insulin degrading enzyme (IDE), myelin basic protein (MBP), necdin (NDN), neuroinflammation, RNA sequencing (RNAseq)

## Abstract

**Background:**

Fragile X syndrome is a monogenic cause of autism spectrum disorder. *Fmr1*-KO mice are a well-known model for the disease, and the model’s behavioral phenotype has been well-characterized. Unfortunately, translational molecular tissue biomarkers for this model remain sparse.

**Methods:**

To identify new molecular biomarkers, we performed a gene sequencing analysis of caudate putamen tissue of *Fmr1*-KO mice at the age of 7 weeks. RNA sequencing analysis revealed 13 genes to be upregulated and 37 genes to be downregulated in the caudate putamen of *Fmr1*-KO mice. The two most upregulated genes were necdin (*Ndn*) and insulin degrading enzyme (*Ide*). Upregulation of *Ndn* and *Ide* genes was confirmed by real-time PCR. NDN protein expression was analyzed in the caudate putamen, hippocampus, and brainstem using immunofluorescent labeling. A co-labeling experiment assessed NDN expression in cholinergic neurons. *Ide* gene upregulation and its effect on insulin levels were evaluated, and protein expression analysis focused on NDN, parvalbumin, and myelin basic protein in the caudate putamen, cortex, and hippocampus.

**Results:**

mRNA levels of *Ndn* and *Ide* and protein levels of NDN and IDE were strongly upregulated in the caudate putamen of *Fmr1*-KO mice. NDN protein expression was also increased in the hippocampus and brainstem, with partial expression observed in cholinergic neurons. Upregulated *Ide* gene expression was associated with reduced insulin levels in *Fmr1*-KO mice.

**Conclusion:**

NDN and IDE emerge as promising candidates for preclinical disease-relevant biomarkers in *Fmr1*-KO mice. Future research needs to demonstrate whether disease-modulating drugs can alter the expression profile of these proteins and ultimately alleviate disease symptoms.

## 1 Introduction

Fragile X syndrome (FXS) is the most inherited form of intellectual disability and a known monogenic cause of autism spectrum disorder (ASD) (Rogers et al., 2001). In the regular form, the *FMR1* gene contains 5–55 triplet repeats of CGG. The mutation of the *FMR1* gene with more than 200 CGG repeats promotes an extensive methylation of the CpG (cytosine 5’ to a guanine, separated by a phosphodiester bond) island, an epigenetic mechanism silencing the gene that encodes the fragile X messenger ribonucleoprotein 1 (FMRP). FMRP regulates expression of several proteins by interacting with up to 4% of the mammalian brain mRNAs (Ascano et al., 2012). FMRP is expressed in various cell types but is mostly present in neurons and testes (Devys et al., 1993; Zhang et al., 2007). Complete loss of FMRP by silencing the *FMR1* gene is known to influence cellular growth, protein synthesis, and energy metabolism via mTOR signaling (Sharma et al., 2010).

Based on the X-linked nature of FXS, phenotypic alterations are observed considerably more often in males than females with approximately 1:7,000 males and 1:11,000 females (Stone et al., 2025). Furthermore, phenotypes are milder in females (Murray et al., 1996; Pembrey et al., 2001). Alterations, such as intellectual disabilities, modified anxiety, hyperactivity, repetitive behavior, social deficits, seizures and hypersensitivity, as well as some physical characteristics such as an elongated face, flat feet, hyperextensible joints, and enlarged testes are observed in male FXS patients (Abbeduto et al., 2004; Hagerman and Hagerman, 2002).

Various animal models of FXS have been developed to not only study FXS, but also ASD. Such models were created in fly, zebrafish, mouse, and rat (McBride et al., 2005; McBride et al., 2012; McTighe et al., 2013; Schneider and Przewłocki, 2005; Tucker et al., 2004). Most importantly, the well-studied *Fmr1*-knock out (KO) mouse model of FXS was initially created by inserting a neomycin cassette in exon 5 (Bakker et al., 1994; Yan et al., 2004). Mice present enlarged testes, altered dendritic spine morphology (Berman et al., 2012; Comery et al., 1997), enhanced metabotropic glutamate receptor activation (Dölen and Bear, 2008), attention deficits and hyperactivity (Ding et al., 2014), excessive repetitive behavior (McNaughton et al., 2008; Veeraragavan et al., 2012), social deficits (Gholizadeh et al., 2014; Pietropaolo et al., 2011; Rotschafer et al., 2012), and thus similar pathologies and symptoms as described for FXS patients (Rogers et al., 2001). Moreover, the intellectual disability observed in FXS patients is mimicked in *Fmr1*-KO mice by hippocampus- and amygdala-dependent memory and cognitive deficits (Arbab et al., 2018; Lorenzini et al., 1996). Although the *Fmr1*-KO model is frequently used for studying FXS and evaluating new drugs against FXS syndromes for their efficacy, molecular tissue biomarkers that might correspond to motor or cognitive deficits remain sparse (Zafarullah and Tassone, 2019). The most obvious are FMR1-related measures such as CGG repeats, methylation, mRNA or FMRP levels which are supposed to account for only a proportion of the FXS phenotypic variability. For adjusting FMR1-related measures, gene therapy approaches would be needed. Additionally, metabotropic glutamate receptors (mGluRs), GABA receptors and BDNF are well-studied dysregulated molecular markers relating to motor deficits in *Fmr1*-KO mice. Unfortunately, treatment of none of these markers resulted in convincing results in FXS patients (Zafarullah and Tassone, 2019).

In our previous *Fmr1*-KO mouse study, we validated a decreased anxiety combined with hyperactivity and impaired communication in this model (Sharghi et al., 2023). Based on these results, we hypothesize that FMRP loss in the CPu leads to specific, measurable transcriptomic alterations. We thus performed a RNA sequencing analysis of caudate putamen (CPu, also referred to as dorsal striatum) tissue of young male *Fmr1*-KO mice. The CPu was chosen as it is well known to be linked to motor control but also control of cognitive processes (Peter et al., 2017). Furthermore, several research groups report that the striatum, the equivalent brain structure to the CPu in humans, displays volumetric changes in ASD as well as FXS patients (Hallahan et al., 2011; Haznedar et al., 2006). As FXS is a neurodevelopmental disorder, early treatment approaches are needed to be able to halt the development of symptoms as early as possible. To account for this need, *Fmr1*-KO mice at the age of only 7 weeks were used to identify early alterations in gene expression. Additionally, this age corresponds to the age at which we previously replicated the autism spectrum disease like behavioral in *Fmr1*-KO mice (Sharghi et al., 2023). Results of the gene sequencing analysis were confirmed by analyzing the expression profile of corresponding mRNA in CPu and proteins in the CPu and other brain regions. With this approach, we aimed to define new tissue biomarkers for preclinical FXS research.

## 2 Materials and methods

### 2.1 Animals

Five male C57BL/6J (B6; #:000664) and five male B6.129P2-*Fmr1*^*TM*1*Cgr*^/J (*Fmr1*-KO; #:003025) were purchased from Jackson Laboratory, Bar Habor, ME, United States (Bakker et al., 1994). Based on vendors information, homozygous female *Fmr1*-KO mice were bred with hemizygous *Fmr1*-KO mice to receive homozygous male *Fmr1*-KO mice (The Jackson Laboratory, 2026). All mice were 6 weeks old at arrival. Animals were housed in groups of 3–5 mice per cage and kept in individually ventilated cages under a 12:12 h dark-light cycle with a temperature of 21 ± 1°C at 40–70% humidity at the AAALAC-accredited animal facility of Scantox Neuro GmbH. Dried pelleted standard rodent chow (Altromin^®^, Lage, Germany) and water were provided *ad libitum*. All animal procedures were conducted in accordance with the Austrian Animal Welfare Act and were approved by the Styrian government, Austria (ABT13-78Jo299-2020).

### 2.2 Tissue sampling

At 7 weeks of age, animals were deeply anesthetized by intraperitoneal injection of 600 mg/kg pentobarbital. Blood was collected via cardiac puncture, stored in EDTA tubes (MiniCollect^®^ K2EDTA potassium ethylenediaminetetraacetic acid) and processed to plasma by centrifugation at 1,000 × g for 15 min at 4°C.

The brain was removed and hemisected. From the left hemisphere, caudate putamen tissue was collected in RNA-later solution (Thermo Fisher, Invitrogen), kept at 4°C overnight, and sent to Lexogen GmbH (Vienna, Austria) on dry ice for RNA extraction and RNA sequencing. The right hemisphere was fixed by immersion in 4% paraformaldehyde in phosphate buffer (pH = 7.4) overnight at 4°C. The right hemisphere was then transferred to 15% sucrose in phosphate buffered saline (PBS) and stored at 4°C for 24 h to provide cryoprotection. On the next day, tissue blocks were transferred to cryomolds (Weckert Labortechnik, Germany), embedded in OCT freezing medium (Leica Biosystems, Germany), frozen in dry ice-cooled isopentane, and stored at -80°C until further analysis.

### 2.3 RNA extraction

Total RNA was extracted by Lexogen GmbH using SPLIT RNA Extraction Kit, Lexogen (PN: 008.48) according to the manufacturer’s guidelines. In brief, tissue was homogenized in 400 μL isolation buffer. Acidic buffer and acidic phenol were added to efficiently separate the genomic DNA. Chloroform was added and phases were cleanly separated using Phase Lock Gel tubes. Further, RNA was purified from the trace amount of phenol and fractionated on a silica column. RNase inhibitor and Enzymatics (PN: Y9240) were added to each sample after elution in 30 μl elution buffer (10 mM Tris-HCl pH 7.0). RNA quantity was measured by UV-Vis spectrophotometry (NanoDrop2000c, Thermo Fisher), RNA integrity was assessed with Agilent 5300 Fragment Analyzer Standard Sensitivity Kit on DNF-471-33-SS Total RNA 15nt method mode.

### 2.4 Library preparation

Sequencing-ready libraries were produced using a QuantSeq 3’ mRNA-Seq Library Prep Kit for Illumina with 500 ng of total RNA from each left CPu as input. Standard procedures were followed as outlined in the respective User Guide (version 113UG227V0110). Quality of the libraries was determined with Agilent 5300 Fragment Analyzer High Sensitivity NGS Fragment Kit on DNF-474-33 - HS NGS Fragment 1–6,000 bp method mode. Concentrations of obtained libraries were quantified using a Qubit dsDNA HS assay (Thermo Fisher). A sequencing-ready pool of indexed libraries was prepared in equimolar ratio according to these quantifications.

The Quant Seq 3′ method for library preparation was used as mRNAs are not fragmented before reverse transcription from the 3’ end. Thus, only one copy of cDNA is produced for each transcript and the number of reads are therefore directly reflecting the number of transcripts of a given gene, regardless of the transcript’s length (Ma et al., 2019; Moll et al., 2014). A direct comparison of whole transcript and 3’ RNA sequencing methods resulted in similar levels of reproducibility between methods (Ma et al., 2019).

### 2.5 RNA sequencing

Sequencing of total RNA from each left CPu was performed on an Illumina NextSeq 2000 system with a NextSeq 1000/2000 P3 Reagent Kit (100 cycles) by Lexogen GmbH, Vienna, Austria. For alignment of trimmed reads to the reference, the STAR (Spliced Transcripts Alignment to a Reference) aligner was used. The method aligns reads directly to a reference genome via the use of a pre-generated index of the reference. The DESeq2 software was used to calculate differential gene expression. Visualization of gene expression results by heat map and principle component analysis plot was prepared by Lexogen GmbH, Vienna, Austria.

### 2.6 Functional enrichment analysis

Functional enrichment analysis was performed by Lexogen GmbH, Vienna, Austria, on the differentially expressed genes (DEGs) identified comparing *Fmr1*-KO mice to C57BL/6J mice, with C57BL/6J mice as baseline, using the Bioconductor package clusterProfiler (v3.x) in R. Gene Ontology (GO) term enrichment analysis was conducted across all three ontology categories (Biological Process, Molecular Function, and Cellular Component), using an adjusted *p*-value (*q*-value) cutoff of 0.05 and a *p*-value cutoff of 0.01. The top 20 enriched GO terms were visualized as bar plots depicting *q*-values and gene counts.

Gene Set Enrichment Analysis (GSEA) was performed by ranking all expressed genes by their log2 fold change values. Gene sets from the Molecular Signatures Database (MSigDB) Hallmark collection (category H) were tested for positional enrichment within the ranked gene list, using a *p*-value cutoff of 0.05. Results were visualized as ridgeline plots.

Reactome pathway enrichment analysis and WikiPathways enrichment analysis were performed using the msigdbr Bioconductor package, which incorporates curated gene sets from the MSigDB database. For both databases, pathway over-representation analysis and GSEA were conducted with a *q*-value cutoff of 0.05 and *p*-value cutoff of 0.01 for over-representation, and a *p*-value cutoff of 0.05 for GSEA. Redundant pathway terms were filtered prior to analysis. All analyses were conducted in R using DESeq2 (v1.18.1) for differential expression with results mapped to mouse (*Mus musculus*) gene annotations.

### 2.7 Polymerase chain reaction (RT-PCR)

mRNA levels of *Ndn* and *Ide* were analyzed by RT-PCR using the same mRNA samples as for RNA sequencing. One microliter of total RNA from each left CPu was reverse transcribed into single stranded cDNA by iScript gDNA Clear cDNA Synthesis Kit according to the manufacturer’s protocol (Bio-Rad Laboratories, Hercules, United States). Quantitative RT-PCR (qPCR) was performed using a Bio-Rad CFX Connect thermo cycler. Takyon No Rox SYBR MasterMix dTTP Blue RT-PCR was used according to manufacturer’s protocol (Eurogentec, Seraing, Belgium) with the primers listed in Table 1. The ΔΔCq method was used to calculate the relative fold-change of mRNA expression normalized to hypoxanthin-guanin-phosphoribosyl-transferase (HPRT) expression. Dilutions of cDNA were prepared and 2 μL primer added to 10 μL of 2x Takyon SYBR mix. *Aqua bidest* was added to a final volume of 20 μL. The qPCR amplification protocol consisted of an activation cycle at 95°C for 3 min, followed by 40 repeat cycles. Denaturation was performed for 3 s at 95°C, annealing at 60°C for 30 s.

**TABLE 1:** List of primers for qPCR analysis.

QuantiTect primer assay (QIAGEN, Germany)		
*Hprt*	Mm_Hprt_1	QT00166758
*Ide*	Mm_Ide_1	QT00122696
*Ndn*	Mm_Ndn_1	QT00290899

*Hprt*, Hypoxanthin-guanin-phosphoribosyl-transferase; *Ide*, Insulin degrading enzyme; *Ndn*, Necdin.

### 2.8 Insulin detection by ELISA

Plasma insulin levels of all animals were quantified by the UltraSensitive Mouse Insulin ELISA Kit (Crystal Chem, United States, Cataloged #90080). Plasma samples were diluted 1:5 in sample diluent and analyzed according to the manufacturer’s instruction. In brief, after dilution, a total sample volume of 100 μl in diluent was added to the pre-coated well and incubated for 2 h at 4°C with gentle agitation. Wells were washed 5 × with 300 μL washing buffer per well. 100 μL of anti-insulin conjugate enzyme was added per well and the plate incubated for 30 min at room temperature (RT). The supernatant was removed, and the plate washed 7 × with 300 μL wash buffer per well. Immediately after washing, 100 μL of enzyme substrate solution was added per well and the plate incubated at RT for 25 min in the dark. 100 μL stop reagent was added per well and after a very short agitation by hand, the plate was read at 450 nm on the Cytation 5 Cell Imaging Multimode Reader (BioTek, United States). Data were evaluated in comparison to the standard provided in the kit as ng/ml medium.

### 2.9 Cryo-cutting

Embedded and frozen brain hemispheres were sagittally cut from the medial to the lateral side using a Cryostat (CryoStar NX70, Thermo scientific, United States). Ten micrometer thick sections were produced from 5 levels -0.54, 1.14, 1.74, 2.34, and 2.94 mm according to the brain atlas of ‘’The Mouse Brain in Stereotaxic Coordinates” (Paxinos and Franklin, 2009). Sections were stored at -20°C until further processing.

### 2.10 Immunofluorescence

Of all study mice, five sagittal brain sections—one of each level – were immunofluorescently labeled. In each of these sections labeling was performed for multiple antigens in the following three combinations: The first combination was labeling for ionized calcium-binding adapter molecule 1 (Iba1), myelin basic protein (MBP), and NeuN; the second for parvalbumin (PV), choline acetyltransferase (ChAT), NDN and tyrosine hydroxylase (TH); and the third for GFAP and PV. All primary and secondary antibodies used are listed in Table 2. Antibodies were diluted either in 1% donkey serum/PBS (Jackson ImmunoResearch, 017-000-121) or M.O.M diluent (BMK-2202, Vector Laboratories, Burlingame, CA, United States). All antibodies except MBP, Iba1, PV, and NeuN required demasking with 1x citrate buffer solution (CBS; Abcam, ab64236). Cryosections were air-dried for at least 45 min and washed with PBS (Lonza, LONBE17-516F) for 10 min. Antigen demasking was performed when required by placing sections in a filled cuvette with 1x CBS buffer. The cuvettes were incubated in a steamer, and the temperature was monitored with a thermometer until reaching 95°C. Cuvettes were then incubated at 95°C for 15 min. Sections were cooled for at least 15 min and then washed 2 × for 5 min with PBS. Autofluorescence was reduced by incubating sections for 4 min in 1 mg/mL sodium borohydride which was dissolved in cooled PBS. Afterwards, sections were washed 3 × for 5 min with PBS. Unspecific binding was blocked with either 10% donkey serum (Jackson ImmunoResearch, 017-000-121) or M.O.M. Blocking reagent (BMK-2202, Vector Laboratories, Burlingame, United States) dissolved in 0.1% Triton-X-100/PBS (Applichem, A1388) for 1 h at RT in a damp chamber. Subsequently, sections were washed 3 × for 5 min with PBS. The mixture of either primary antibodies or the dilution solution (negative control) was added to the sections which were then incubated in a damp chamber at 4°C overnight. On the next day, sections were washed 3 × for 5 min with PBS. The mixture of secondary antibodies conjugated to a fluorophore was added to the sections and incubated for 1 h at RT in the dark. Sections were washed 3 × for 5 min with PBS. Nuclei were stained with DAPI working solution (1 μl 4‘,6-diamidin-2-phenylindole (DAPI, A1001.0025, AppliChem GmbH, Darmstadt, Germany) diluted in 10 mL ddH_2_O and subsequently 10 mL PBS) for 15 min at RT in the dark. Sections were washed 2 × for 5 min with PBS and subsequently 1 × 5 min with ddH2O. Sections were covered with Mowiol (81381, Sigma-Aldrich, St. Louis, United States) and coverslips using a Leica CV5030 cover slipper. Immunofluorescence images were captured with the section scanner Zeiss AxioScan.Z1 (Carl Zeiss Microscopy, United States) at 10× magnification. The exposure time was kept constant for all sections within an incubation. Representative negative control images with primary antibody omitted for validation of antibody specificity are shown in Supplementary Figure 1.

**TABLE 2:** List of antibodies for histological analyses.

Antibody	Primary/secondary	Manufacturer	Item number	Host	Dilution
IBA1/AIF1	Primary	Synaptic Systems	234013	Rabbit	1:1,000
MBP		Abcam	ab134018	Chicken	1:500
NeuN		Synaptic Systems	266004	Guinea pig	1:1,000
pv		Swant	PVG-213	Goat	1:3,000
ChAT		Merck Millipore	AB144P-200UL	Goat	1:300
NDN		Abcam	ab227908	Rabbit	1:600
TH		Synaptic Systems	213104	Guinea pig	1:5,000
GFAP		Abcam	ab53554	Goat	1:2,000
PV		Swant	PV235	Mouse	1:1,000
Alexa Fluor 750	Secondary	Abcam	ab175728	Donkey	1:500
Alexa Fluor 647		Jackson ImmunoResearch	703-603-155		
Alexa Fluro 488		Jackson ImmunoResearch	706-545-148		
DyLight 550		Abcam	ab96936		
DyLight 650		Abcam	ab96922		
Alexa Fluor 750		Jackson ImmunoResearch	706-545-148		

For each primary and secondary antibody, the manufacturer, item number, host and used dilution factor is mentioned.

### 2.11 Quantitative evaluations

Images were processed with the Image-Pro 10 software (Media Cybernetics, United States). The region of interest (ROI) was identified by manual delineation of the cortex, hippocampus, caudate putamen, brain stem, and corpus callosum. However, the ROIs were different in various incubations depending on the target. An additional ROI was set to exclude wrinkles, air bubbles, or any other artifact interfering with the first ROI, if necessary. For each antibody, positive objects were identified by thresholding, and the results were then filtered for size and shape factors to focus on appropriate objects (e.g., neuronal somata, astrocytes). Activated microglia were identified by applying a minimum object size of 40 μm^2^ paired with very intense fluorescence signal intensity to separate these from non-activated Iba1+ cells. Fluorescent signals were evaluated within each defined ROI for each antibody target within the respective channel. The mean object size (size of above-threshold immunoreactive objects), mean object intensity (average brightness of pixels of above-threshold immunoreactive objects, indicating differences in the cellular expression level of target proteins), object density (number of objects per mm^2^; number of above-threshold immunoreactive objects normalized to the size of the target area; detecting changes in neuronal density), and percentage of the total immunoreactive (IR) area (percentage of the ROI that is covered by above-threshold immunoreactive objects) were quantified and the group mean average was calculated. Co-expression was evaluated by detecting all NDN+ somata and all ChAT+ somata in a brain area, then calculating the percentages of co-expressing cells based on the total cell count for each marker in this brain area (Supplementary Table 1).

### 2.12 Statistical analysis

All statistical analyses and graph preparation were performed by GraphPad Prism*TM* 9.2 software (GraphPad Software Inc., United States). Outliers were detected by the Grubb’s outlier test and excluded. Outliers are explicitly mentioned in the corresponding figure legend. All groups were evaluated for normal distribution and compared by unpaired *t*-tests corrected for multiple testing (Benjamini Hochberg false discovery rate; Supplementary Tables 2–7 for detailed results) Data are shown as bar graphs in combination with dot plots; mean ± SEM; Statistical significances are displayed as *p < 0.05; **p < 0.01; ***p < 0.001. See figure legends for details.

## 3 Results

### 3.1 RNA sequencing analysis

Total RNA was extracted from frozen caudate putamen tissue and the quality evaluated. RNA concentrations ranged from 164.7 to 450.6 ng/μL. The A260/A280 ratio ranged from 2.03 to 2.06 while the A260/A230 ratio ranged from 1.79 to 2.14. RNA quality was thus rated as sufficient for gene expression analysis. The differential expression analysis identified 50 significantly dysregulated genes in *Fmr1-KO* mice (37 downregulated, 13 upregulated, adjusted *p* < 0.10), out of 24,298 total expressed genes in the caudate putamen of *Fmr1*-KO compared to C57BL/6J mice. The most significantly downregulated gene in *Fmr1*-KO mice was Fmr1 (log2 fold change (log2FC) = 1.13, adj. *p* = 4.1 × 10^–30^), serving as an important internal positive control validating the experimental model.

For a full list of genes, log2 fold change, *p*-values and adjusted *p*-values as well as visualization of gene expression results as heat map and principle component analysis (PCA) see Supplementary Table 8 and Supplementary Figure 2, respectively. The 13 significantly upregulated genes were *Ndn, Ide, Fgfbp3, Rab39b, Car10, Snrpc, Fcrls, Sst, Etv5, Aqp4, Npy, Ywhag* and *Bex2.* The 20 most downregulated genes were *Fmr1, Gstm1, Rida, Apod, Gm5441, Pitpnm3, Arhgdib, Abi3bp, Lyz2, Mafa, Cryzl2, Rarres2, Cd59a, Amz1, Ano2, Tppp3, Tns1, Pdzd2, Ptgds*, and *Pipox* (Table 3).

**TABLE 3:** Top 20 differentially expressed genes in the caudate putamen of *Fmr1*-KO mice compared to C57BL/6J mice.

In Fmr1-KO mice upregulated genes				In Fmr1-KO mice downregulated genes			
Gene ID	Gene	log2Fold change	Adjusted *p*-value	Gene ID	Gene	log2Fold change	Adjusted *p*-value
***ENSMUSG00000033585***	*Ndn*	**0.5**	< 0.001	***ENSMUSG00000000838***	*Fmr1*	**-1.13**	< 0.001
***ENSMUSG00000056999***	*Ide*	**0.70**	< 0.001	***ENSMUSG00000058135***	*Gstm1*	**-0.50**	0.001
***ENSMUSG00000047632***	*Fgfbp3*	0.44	< 0.01	***ENSMUSG00000022323***	*Rida*	**-**0.45	0.01
***ENSMUSG00000031202***	*Rab39b*	0.44	< 0.01	***ENSMUSG00000022548***	*Apod*	**-**0.44	< 0.001
***ENSMUSG00000056158***	*Car10*	0.37	0.08	***ENSMUSG00000110020***	*Gm45441*	**-**0.43	0.018
***ENSMUSG00000024217***	*Snrpc*	0.37	0.06	***ENSMUSG00000040543***	*Pitpnm3*	**-**0.43	0.008
***ENSMUSG00000015852***	*Fcrls*	0.36	0.07	***ENSMUSG00000030220***	*Arhgdib*	**-**0.40	0.020
***ENSMUSG00000004366***	*Sst*	0.35	< 0.001	***ENSMUSG00000035258***	*Abi3bp*	**-**0.40	0.04
***ENSMUSG00000013089***	*Etv5*	0.31	0.07	***ENSMUSG00000069516***	*Lyz2*	**-**0.39	0.05
***ENSMUSG00000024411***	*Aqp4*	0.26	0.09	***ENSMUSG00000047591***	*Mafa*	**-**0.39	0.05
***ENSMUSG00000029819***	*Npy*	0.24	0.06	***ENSMUSG00000033488***	*Cryzl2*	**-**0.38	0.05
***ENSMUSG00000051391***	*Ywhag*	0.22	0.02	***ENSMUSG00000009281***	*Rarres2*	**-**0.38	0.06
***ENSMUSG00000042750***	*Bex2*	0.20	0.06	***ENSMUSG00000032679***	*Cd59a*	**-**0.38	0.02
				***ENSMUSG00000050022***	*Amz1*	**-**0.38	0.06
				***ENSMUSG00000038115***	*Ano2*	**-**0.38	0.06
				***ENSMUSG00000014846***	*Tppp3*	**-**0.37	0.05
				***ENSMUSG00000055322***	*Tns1*	**-**0.37	0.004
				***ENSMUSG00000022197***	*Pdzd2*	**-**0.36	0.02
				***ENSMUSG00000015090***	*Ptgds*	**-**0.35	0.02
				***ENSMUSG00000017453***	*Pipox*	**-**0.35	0.05

Up- and down-regulation was determined by RNA sequencing. Genes with the largest log2-fold change are shown in bold. Only significantly changed genes are shown.

### 3.2 Ndn expression in *Fmr1*-KO mice

The *Ndn* gene was the strongest upregulated gene in *Fmr1-KO* mice, and we therefore evaluated its expression in more detail. Quantification of *Ndn* mRNA levels by qPCR showed a 2-fold increase of *Ndn* levels in the caudate putamen of *Fmr1*-KO mice compared to C57BL/6J mice and therefore confirming gene sequencing results (Figure 1A, *p* = < 0.001). Further quantification of NDN protein levels in the CPu by immunofluorescent labeling resulted in a significantly increased NDN immunoreactive (IR) area (*p* = 0.004) as well as object size (*p* = 0.006) of *Fmr1*-KO mice compared to C57BL/6J mice (Figures 1B–D). The NDN-positive object density and object intensity were not altered in *Fmr1*-KO mice compared to C57BL/6J mice (Supplementary Figures 3A,B).

**FIGURE 1:**
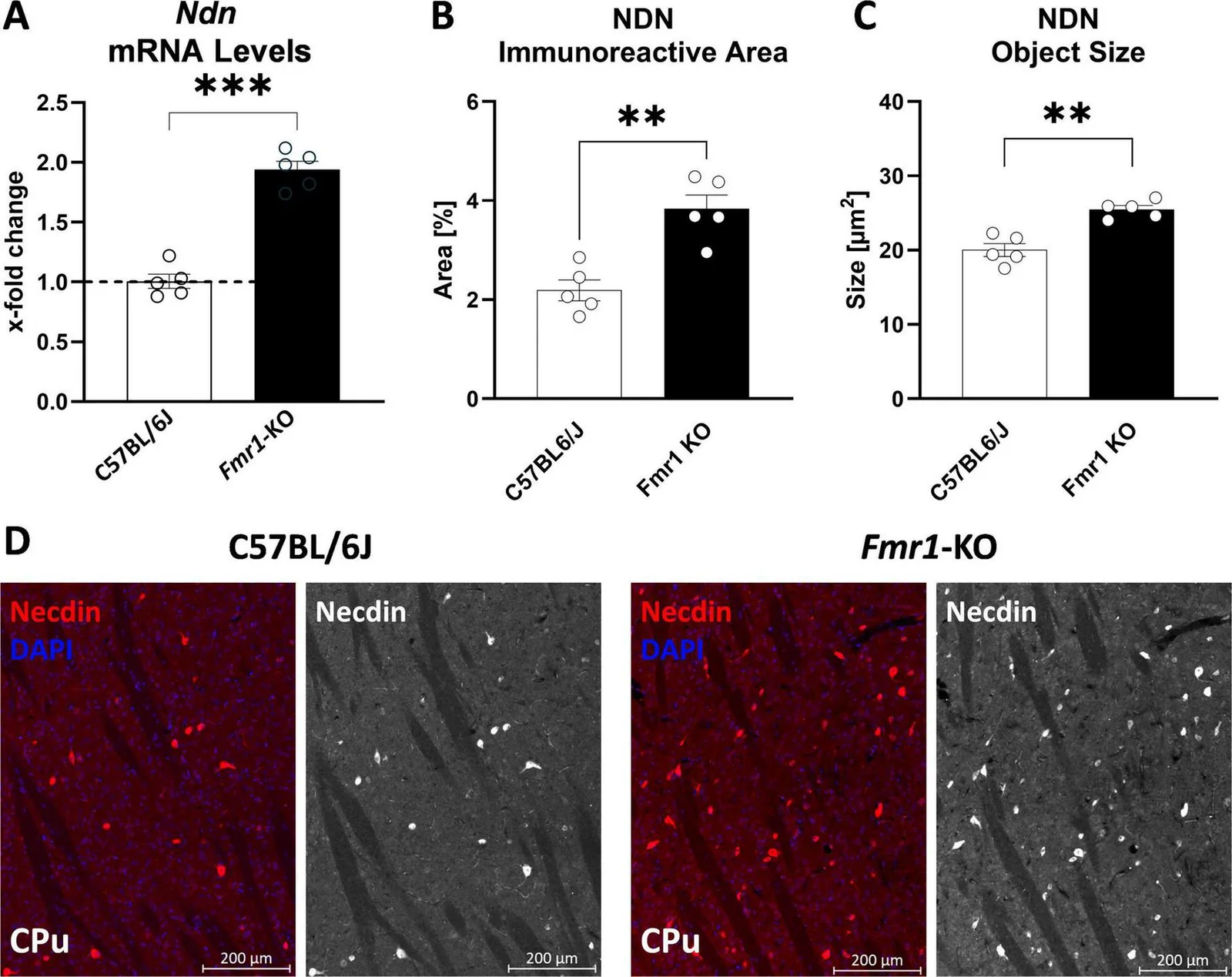
Quantification of *Ndn* mRNA and NDN protein expression in the CPu of *Fmr1*-KO and C57BL/6J mice. **(A)** Necdin (*Ndn*) mRNA expression levels in the caudate putamen (CPu) of 7 weeks old *Fmr1*-KO mice as analyzed by qPCR; hypoxanthine-guanine phosphoribosyltransferase (HPRT) was used as housekeeping gene. NDN protein levels were quantified by immunofluorescence analysis of the NDN immunoreactive (IR) area in percent **(B)** and NDN object size in μm^2^ **(C)** in the CPu. **(A–C)** *n* = 5 per group; mean ± SEM; unpaired *t*-test; **p < 0.01; ***p < 0.001. **(D)** Representative images of immunofluorescent labeling of NDN (red) and DAPI (blue, counterstaining of nuclei) in the CPu of *Fmr1*-KO and C57BL/6J mice. DAPI, 4’,6-diamidin-2-phenylindole.

In the next step, NDN expression was evaluated in the hippocampus and brainstem. Results show that the NDN IR area (*p* = 0.03) and object size (*p* = 0.012) were significantly increased in the hippocampus of *Fmr1*-KO mice compared to C57BL/6J mice but not as strong as in the CPu (Figures 2A, B, D). Evaluation of the NDN expression in the brainstem of *Fmr1*-KO mice showed no differences in the IR area but in the object size (*p* = 0.012) compared to C57BL/6J mice (Figures 2C–F). The NDN-positive object density and object intensity in the hippocampus and brainstem of *Fmr1*-KO mice were not altered compared to C57BL/6J mice (Supplementary Figure 4). Further quantitative analyses revealed significant differences also in other brain regions such as the ventral thalamus (data not shown).

**FIGURE 2:**
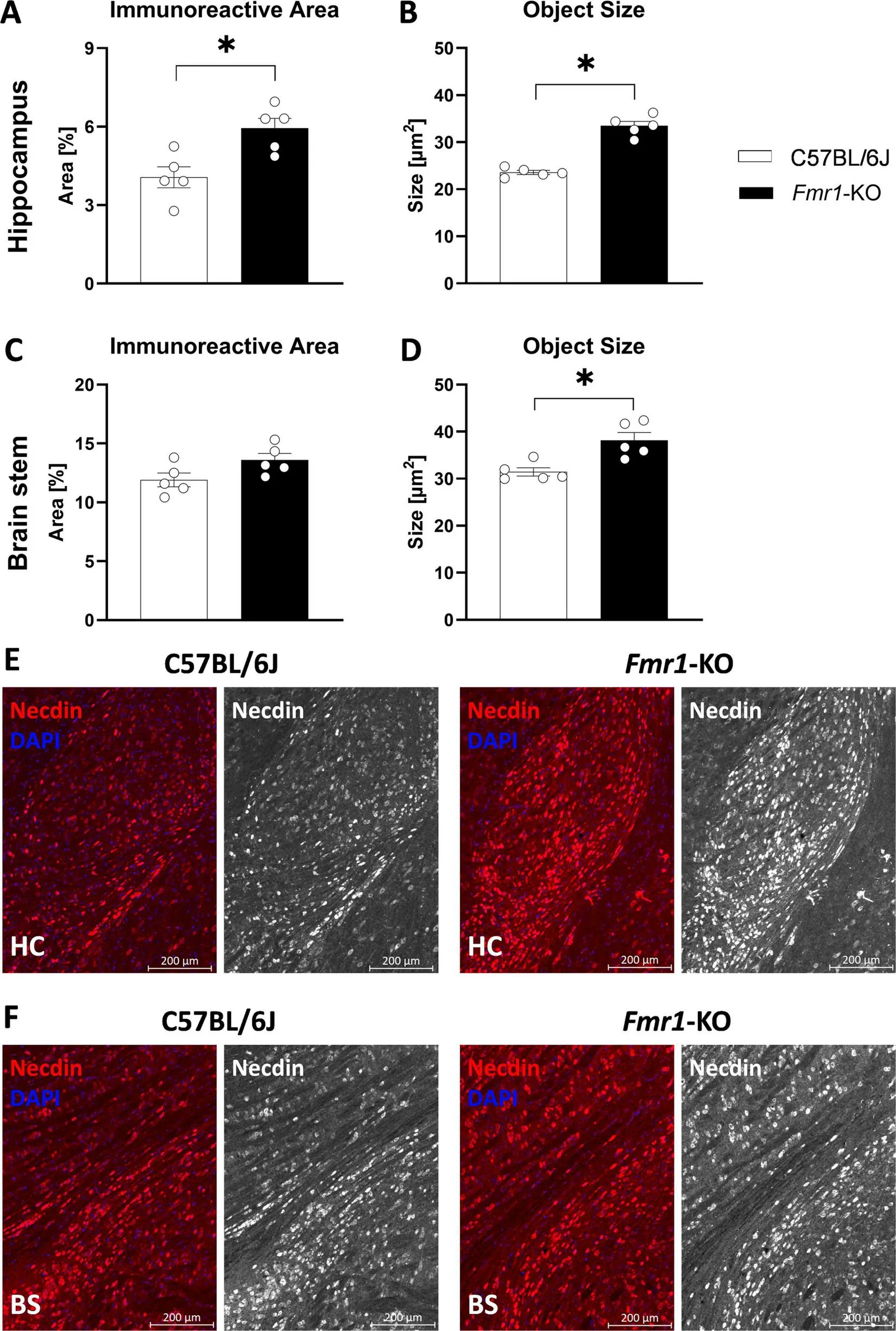
Quantification of NDN protein expression in the hippocampus and brainstem of *Fmr1*-KO and C57BL/6J mice. Quantification of necdin (NDN) immunoreactive (IR) area in percent **(A,D)** and NDN object size in μm^2^ **(B,E)** in the hippocampus **(A,B)** and brainstem **(D,E)**. **(A,B,D,E)** *n* = 5 per group; mean ± SEM; unpaired *t*-test; *p < 0.05. **(C,F)** Representative images of NDN (red) and DAPI (blue, counterstaining of nuclei), labeling in the hippocampus (HC, **C**) and brainstem (BS, **F**) of *Fmr1*-KO and C57BL/6J mice. DAPI, 4’,6-diamidin-2-phenylindole.

### 3.3 NDN expression in cholinergic neurons

To evaluate in which cell types NDN is expressed, a quadruple immunofluorescent labeling was performed to label for NDN, TH as marker of catecholaminergic neurons, PV as marker of GABAergic interneurons, and ChAT as marker of cholinergic neurons in the caudate putamen. The labeling showed NDN expression in some ChAT-positive cells, while TH and PV expression did not overlap with NDN expression in C57BL/6J as well as *Fmr1*-KO mice. However, co-expression of NDN and ChAT was only partial and differed between brain areas (Figure 3). Basic quantification of the NDN and ChAT co-expression pattern in the caudate putamen resulted in significant changes between *Fmr1*-KO and C57BL/6J mice (Figures 3A,B). The proportion of NDN+ cells that are also ChAT+ was reduced in *Fmr1*-KO mice (*p* = 0.025), while the proportion of ChAT+ cells that are also NDN+ increased (*p* = 0.006). The co-expression pattern in the hippocampus and brain stem did not significantly change between groups (Figures 3C–F).

**FIGURE 3:**
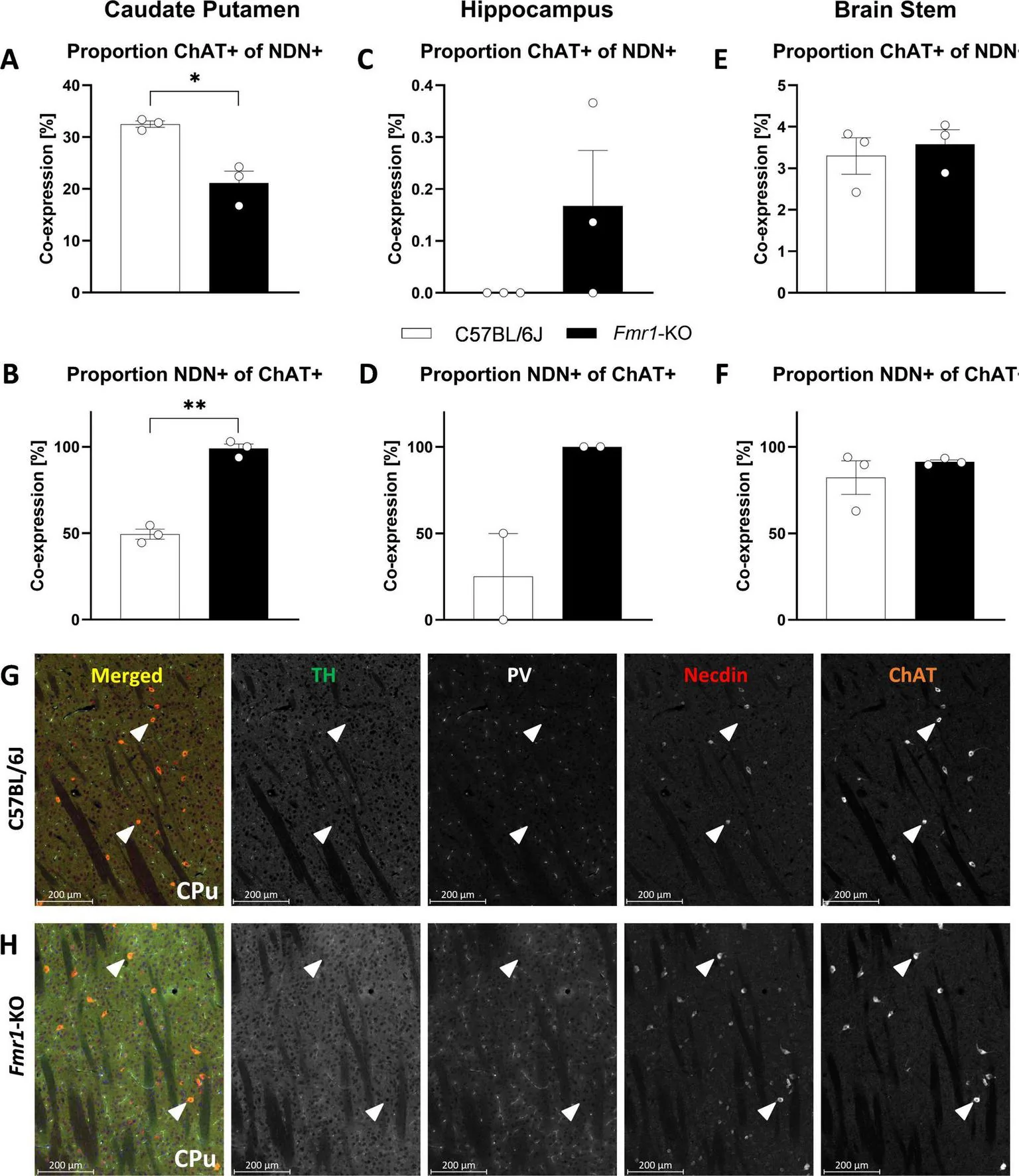
NDN expression in cholinergic neurons. Quantification of the proportion of ChAT+ cells of NDN+ cells in the caudate putamen **(A)**, hippocampus **(C)** and brain stem **(E)** of *Fmr1*-KO compared to C57BL/6J mice. Quantification of the proportion of NDN+ cells of ChAT+ cells in the caudate putamen **(B)**, hippocampus **(D)** and brain stem **(F)** of *Fmr1*-KO compared to C57BL/6J mice. (**A–F**) *n* = 3 per group; mean ± SEM; unpaired *t*-test; *p < 0.05; **p < 0.01. Representative images of tyrosine hydroxylase (TH, green), parvalbumin (PV, white), necdin (red), and choline acetyltransferase (ChAT, orange) labeling in the caudate putamen (CPu) of 7 weeks old C57BL/6J **(G)** and *Fmr1*-KO **(H)** mice. DAPI (blue) was used to counterstain nuclei. White arrowheads show cells expressing NDN and ChAT but not TH or PV. DAPI, 4’,6-diamidin-2-phenylindole.

As we did not observe NDN expression in PV-positive cells, the PV IR area and object density was neither altered in the cortex (Figures 4A,D) nor in the CPu (Figures 4B,E) and hippocampus (Figures 4C,F).

**FIGURE 4:**
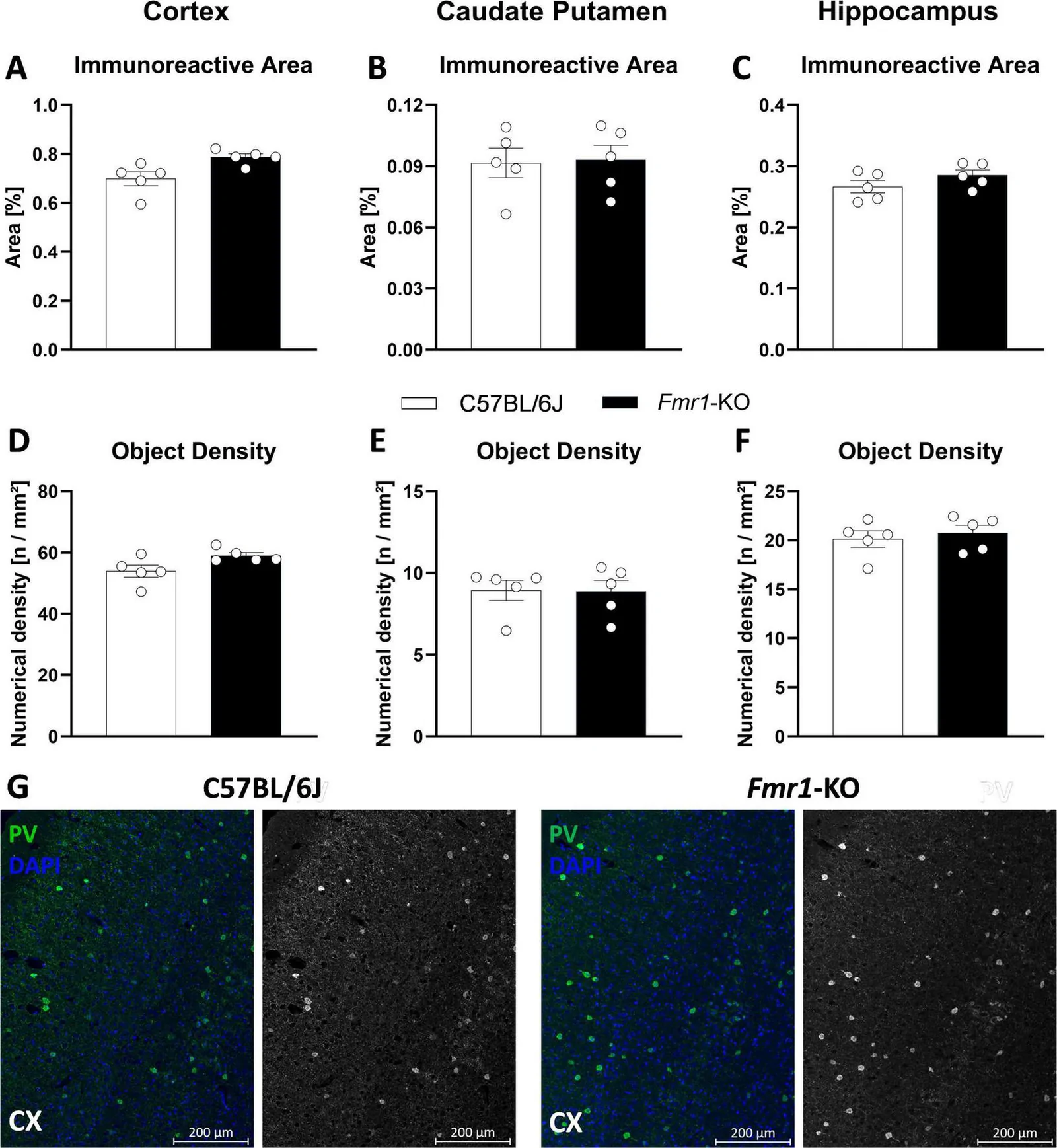
Quantification of PV-positive cells in *Fmr1*-KO and C57BL/6J mice. Parvalbumin (PV) immunoreactive (IR) area in percent **(A–C)** and PV object density in n/mm^2^ **(D–F)** in the caudate putamen **(A,D)**, cortex (CX; **B**,**E**), and hippocampus **(C,F)**. *n* = 5 per group; mean ± SEM; unpaired *t*-test. **(G)** Representative images of PV (green) labeling in the CX of C57BL/6J and *Fmr1*-KO mice. DAPI (blue) was used to counterstain nuclei. DAPI, 4’,6-diamidin-2-phenylindole.

### 3.4 IDE expression *Fmr1*-KO mice

The second most upregulated gene in the gene expression analysis of CPu tissue of *Fmr1*-KO mice compared to C57BL/6J mice was the IDE. To confirm these results, we quantified *Ide* mRNA levels in the CPu by qPCR resulting in 2-fold increased *Ide* mRNA levels in the CPu of *Fmr1*-KO mice compared to C57BL/6J mice (Figure 5A, *p* = < 0.001). As the IDE protein is essential for appropriate insulin signaling, we quantified insulin in the plasma of *Fmr1*-KO mice. Results show that insulin was significantly decreased in the plasma of *Fmr1*-KO mice compared to C57BL/6J mice (Figure 5B, *p* = 0.05).

**FIGURE 5:**
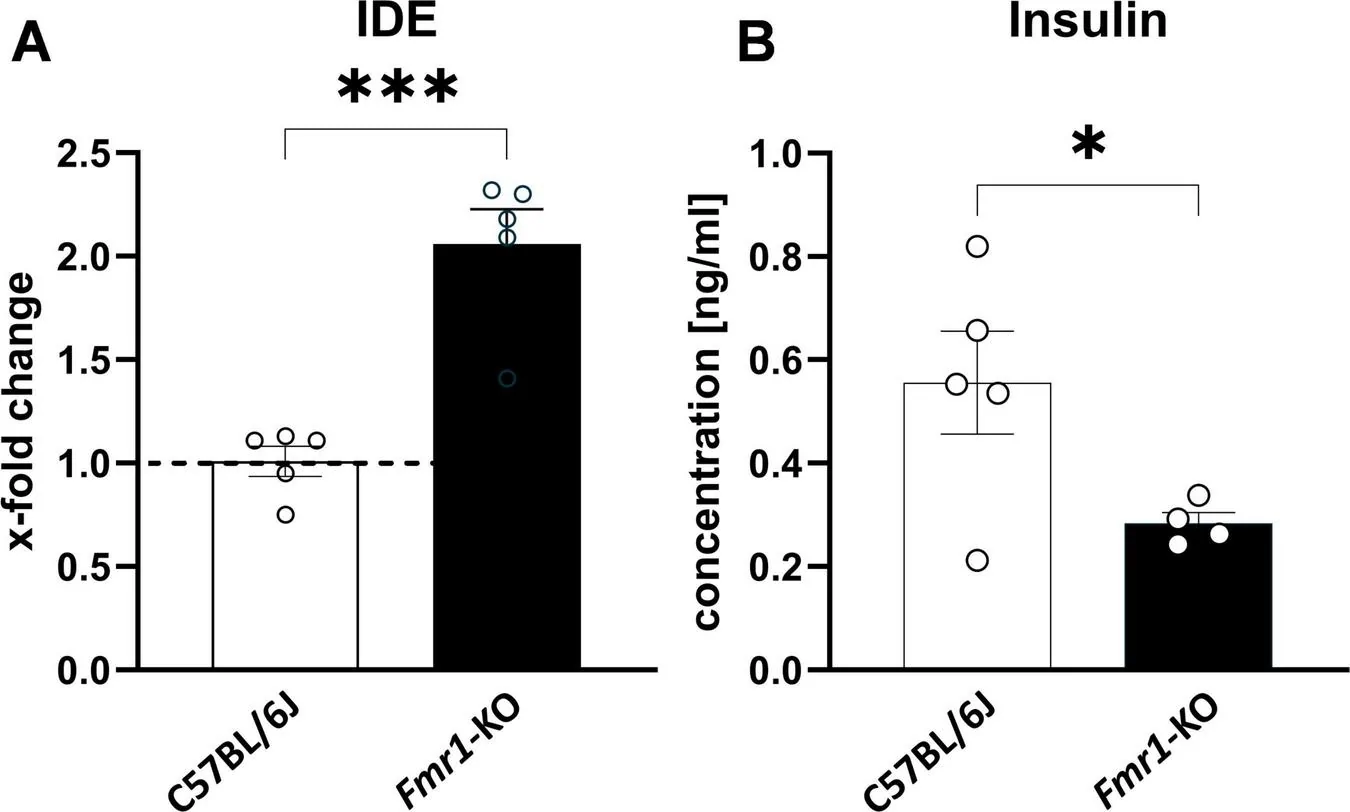
Quantification of *Ide* mRNA levels in the caudate putamen and insulin levels in plasma. **(A)** mRNA expression levels of the insulin degrading enzyme (*Ide*) in the caudate putamen of 7 weeks old *Fmr1*-KO mice normalized to C57BL/6J mice. HPRT was used as a housekeeping gene. **(B)** Quantification of insulin levels in ng/ml plasma. One outlier was detected in *Fmr1*-KO mice by Grubb’s test (α = 0.05) and removed. *n* = 4–5 per group; mean ± SEM; unpaired *t*-test; *p < 0.05, ***p < 0.001. HPRT, hypoxanthine-guanine phosphoribosyl transferase.

### 3.5 MBP expression in *Fmr1*-KO mice

Based on the results of the gene sequencing analysis, *Mbp* gene expression is downregulated in the CPu of *Fmr1*-KO mice compared to C57BL/6J mice (Supplementary Table 8). To evaluate if this downregulation can also be observed on protein level, MBP protein levels were quantitatively evaluated by immunofluorescent labeling. Results show no differences in protein MBP levels in the caudate putamen, cortex, hippocampus and corpus callosum of *Fmr1*-KO and C57BL/6J mice (Figure 6). Neither in IR area, object density, object intensity nor object size.

**FIGURE 6:**
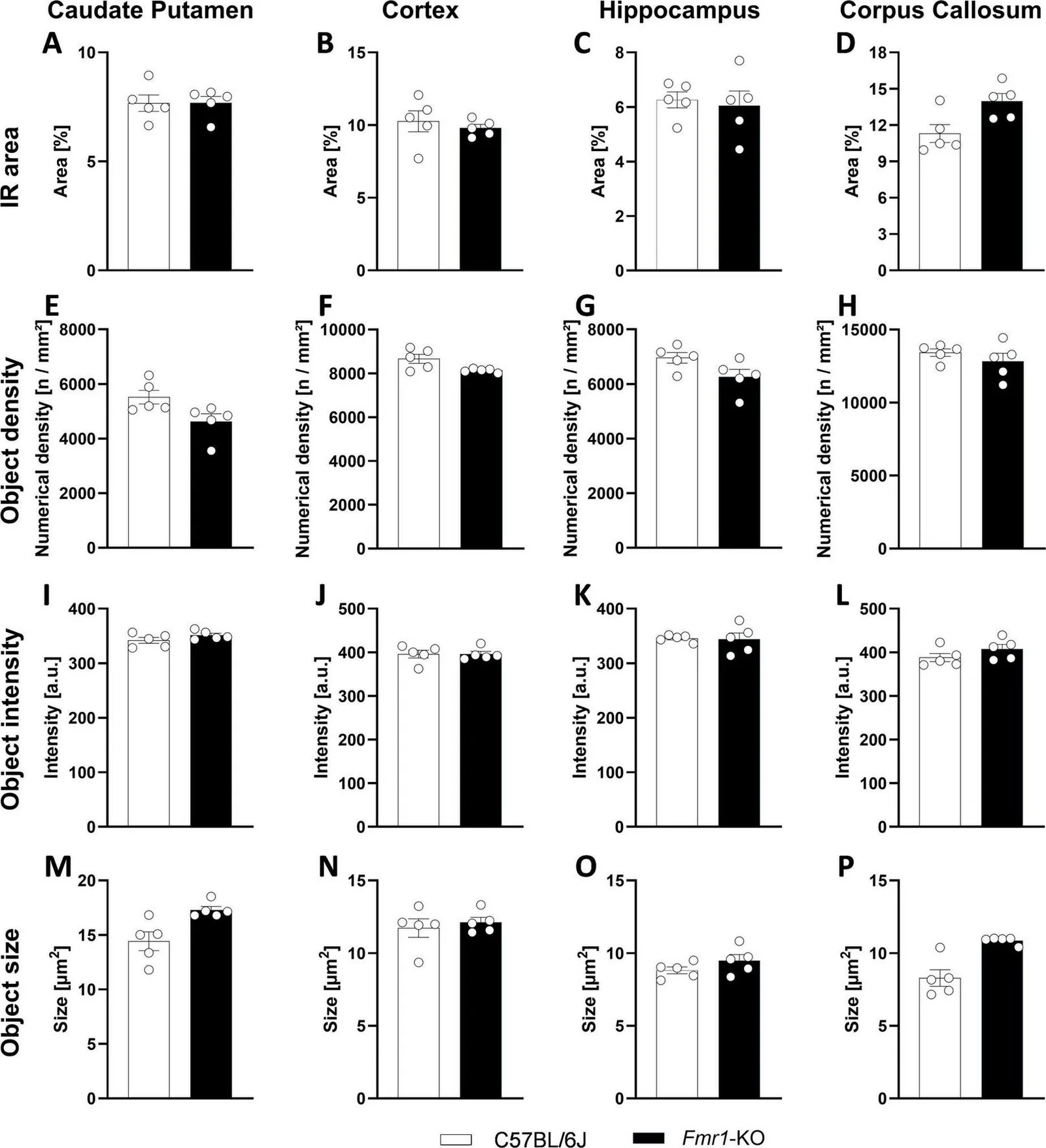
Quantification of MBP in different brain regions of *Fmr1*-KO and C57BL/6J mice. **(A–D)** Myelin basic protein (MBP) immunoreactive (IR) area in percent, **(E–H)** object density in n/mm^2^, **(I–L)** Object intensity in arbitrary units (a.u.), and **(M–P)** object size in μm^2^ in the caudate putamen **(A,E,I,M)**, cortex **(B,F,J,N)**, hippocampus **(C,G,K,O)**, and corpus callosum **(D,H,L,P)**. *n* = 5 per group; mean ± SEM; unpaired *t*-test.

### 3.6 Pathway-level analysis of differentially expressed genes

To move beyond individual gene-level observations and provide a broader biological interpretation of the transcriptomic changes in the *Fmr1*-KO mouse model, we performed comprehensive functional enrichment analyses including GO (Gene Ontology) term enrichment, GSEA (Gene Set Enrichment Analysis), Reactome pathway analysis, and WikiPathways analyses. GO term enrichment analysis of the DEGs revealed enrichment in biological processes to myelin sheath and its structural and constituent (Figure 7A). GSEA analysis shows enrichment in inflammatory processes by NTF-α signaling via NfκB (Figure 7B). The Reactome Pathway Term Enrichment Analysis of the top 20 dysregulated genes revealed no significant pathways (data not shown). WikiPathways term enrichment analysis validates GO term results by indicating enrichment in myelin-regulated biological processes. WikiPathways gene set enrichment analysis further shows enrichment in calcium regulation in cardiac cells, the inflammation related Nrf2 pathway, and oxidative stress pathways. Reactome gene set enrichment analysis eventually corroborates observations, highlighting dysregulation in pathways related to synaptic function and neuronal signaling, renal function, glycosylation, rRNA processing, and protein synthesis (Figures 7C–E).

**FIGURE 7:**
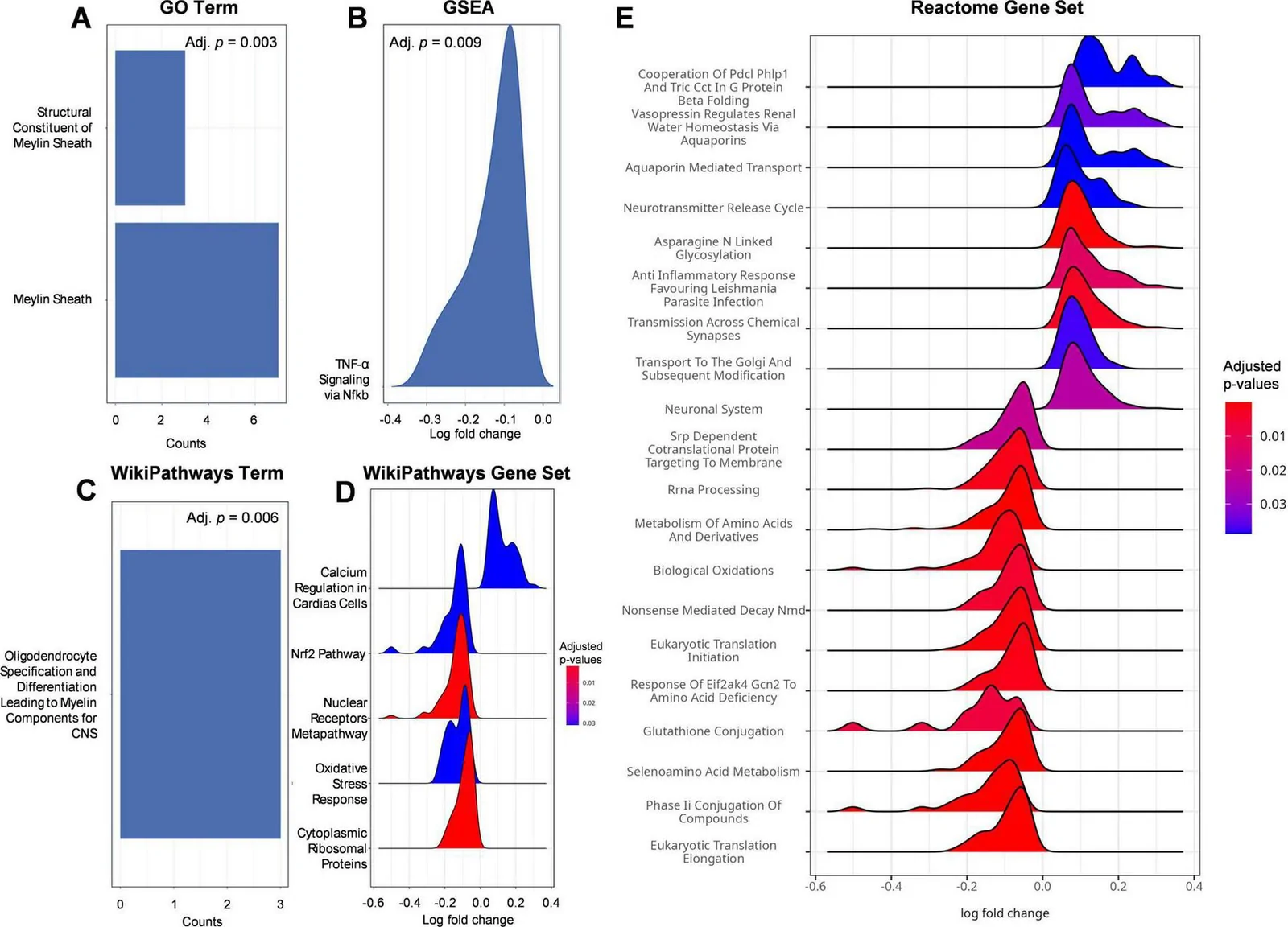
Enrichment Analysis Plots for the top 20 dysregulated genes in *Fmr1*-KO mice compared to C57BL/6J mice. **(A)** GO term enrichment analysis plot. **(B)** Gene set enrichment analysis plot. **(C)** WikiPathways term enrichment analysis plot. **(D)** WikiPathways gene set analysis plot. **(E)** Reactome gene set enrichment analysis plot. Adjusted *p*-values are indicated for each plot.

### 3.7 Neuroinflammation in *Fmr1*-KO mice

As neuroinflammation is a repeatedly reported pathology in *Fmr1*-KO mice, we evaluated astrocytosis and activated microglia by quantifying the GFAP and Iba1 immunoreactive signal after immunofluorescent labeling in the brain of *Fmr1*-KO mice. Changes in the GFAP IR area were found in the CPu and hippocampus of *Fmr1*-KO mice compared to C57BL/6J mice (Supplementary Figures 5A, *p* = 0.45; Supplementary Figures 5C, *p* = 0.018) while in the cortex no changes could be shown (Supplementary Figure 5B). Further analysis of the GFAP-positive object size resulted in no significant changes in the CPu (Supplementary Figure 5D), but a significant increase in the cortex and hippocampus (Supplementary Figure 5E, *p* = 0.012; Supplementary Figure 5F, *p* = 0.006). Parallel analysis of activated microglia by Iba1 labeling resulted in an significantly increased Iba1 IR area and object density in the CPu of *Fmr1*-KO mice compared to C57BL/6J mice (Supplementary Figures 6B,E, *p* = 0.006, *p* = 0.003, respectively) while for both parameters no changes could be observed in the cortex or hippocampus (Supplementary Figures 6A,C,D,F).

## 4 Discussion

mRNA sequencing of *Fmr1*-KO mouse CPu tissue at the age of 7 weeks returned 13 genes to be significantly upregulated and 37 genes to be significantly downregulated. Except for the *Fmr1 gene*, none of these genes were found to be altered in previous gene expression analyses in cortical and hippocampal tissue (Prilutsky et al., 2015) or dorsal striatal tissue of *Fmr1*-KO brain (McCarthy et al., 2024). Not surprisingly we found the *Fmr1* gene to be the strongest downregulated gene - providing good proof of concept. Sequencing further resulted in two mRNA candidates that were strongly upregulated, *Ndn* and *Ide*.

*Ndn* was already shown to be upregulated in adult neural stem cells of *Fmr1*-KO mice (Liu et al., 2018) by transcriptomic analysis. Liu and colleagues concluded from their study that the transcription factor NDN is repressed by FMRP, a possibility that would also be compatible with our results, although a direct interaction between NDN and FMRP still needs to be explored. Functional analyses have shown that NDN regulates several transcriptional and post-transcriptional pathways essential for neural differentiation (Liu et al., 2018). The human *NDN* gene locates in a chromosomal region involved in the pathogenesis of Prader-Willi syndrome that in turn is caused by the paternal gene locus (Jay et al., 1997). In a mouse model with disrupted *Ndn* function, typical symptoms of the Prader-Willi syndrome, such as skin scraping/grooming activity and spatial learning and memory deficits, were thus observed (Muscatelli et al., 2000). Otherwise, excessive amounts of NDN are shown to be a driver of paternal derived chromosome 15q11-q13 duplication (Tamada et al., 2021). This is of immense relevance, as chromosome 15q11-q13 duplication displays one of the most cytogenetically abnormal copy number variations for ASD (Pinto et al., 2014). As excess levels of NDN cause typical ASD pathologies, such as enhanced spine formation and hyperexcitability of cortical pyramidal neurons as well as behavioral deficits (Tamada et al., 2021), additional research is needed to elucidate the impact of NDN on the pathophysiology of *Fmr1*-KO mice and fragile X syndrome patients.

Our NDN expression analyses in different brain regions and neuronal cell types of *Fmr1*-KO mice provide important new information on NDN, as it is shown to be upregulated in several brain regions relevant for ASD pathology, such as CPu and hippocampus. Furthermore, quadruple labeling of NDN with markers of catecholaminergic, GABAergic, and cholinergic cells showed that NDN seems to be solely expressed in a subgroup of cholinergic neurons in *Fmr1*-KO mice. This result is specifically interesting, as reduced NDN levels were demonstrated to result in a decrease of dopaminergic neurons in the substantia nigra of adult mice (Hasegawa et al., 2016) while NDN expression promotes differentiation and migration of GABAergic neurons during development (Kuwajima et al., 2006). Expression of NDN in cholinergic neurons was to our knowledge so far not described.

While our results indicate that NDN might serve as potential preclinical tissue biomarker, translation of these results into the clinic will be challenging, as NDN is so far shown to be solely expressed intracellularly. Current data do not provide evidence for NDN secretion into the blood or CSF which would allow *in vivo* analysis of the protein in the clinic (Yoshikawa, 2021).

*Ide* was the second strongest upregulated gene in our gene expression analysis. As Liu and colleagues did not observe upregulated *Ide* levels in *Fmr1*-KO adult neural stem cells (Liu et al., 2018) this effect seems to be cell type specific. Additional analyses are needed to untangle in which cell types IDE is upregulated. Upregulated *Ide* levels coincided with reduced plasma insulin levels, suggesting that FMRP might influence insulin signaling in *Fmr1*-KO mice, although further research is needed to validate causal relationship. In 2019, Leboucher and colleagues observed reduced insulin levels in the conditional *Fmr1*-KO2 mouse model (Leboucher et al., 2019). The team evaluated the effect of FMRP on the glucose metabolism more closely and observed an enhanced glucose tolerance and insulin response in these mice. Further analysis of serum from FXS patients resulted in similarly reduced insulin levels as in the analyzed mouse model (Leboucher et al., 2019). Although altered insulin metabolism across *Fmr1* transgenic mouse models and patients thus seems to be a highly translational pathology that provides an easily accessible and measurable readout to evaluate the effect of FXS therapy approaches, additional research is needed to uncover if or how FMRP impacts insulin signaling.

Aside from our results about IDE expression in various brain regions of *Fmr1*-KO mice which will enable to use IDE as tissue marker in preclinical studies, others were able to measure IDE protein in the cerebrospinal fluid of mice and men (Son et al., 2016) and thus defining IDE as biomarker that potentially could be translated to clinical research (Son et al., 2016). Interestingly, Son and colleagues showed in this publication that the amyloid-β peptide is able to reduce IDE levels in the CSF of mice and that IDE levels are decreased in the CSF of a mouse model of Alzheimer’s disease (AD) and patients with a diagnosed AD (Son et al., 2016). As the amyloid-β protein precursor is already well-known to be dysregulated in *Fmr1*-KO mice (Westmark, 2019), further research is needed to untangle the underlying signaling pathways.

As FMRP is under physiological conditions prominently expressed in GABAergic neurons, many researchers have focused on investigating the role of FMRP in GABAergic neuronal populations (Olmos-Serrano et al., 2010; Paluszkiewicz et al., 2011; Reyes et al., 2020; Sabanov et al., 2017; Yang et al., 2022). *Fmr1*-KO mice show defects in inhibitory GABAergic circuitry (Cea-Del Rio and Huntsman, 2014; Paluszkiewicz et al., 2011) that can even impact on behavioral abnormalities (Lee et al., 2019). As it was previously shown that the number of PV-positive (GABAergic) neurons in the somatosensory cortical layers II/III/IV of 1 year old *Fmr1*-KO mice are increased (Selby et al., 2007), this result does not match our findings. In total, these observations could suggest a contribution of FMRP in cortical lamination and cellular positioning in *Fmr1*-KO mice during development (Castrén, 2016; Moro et al., 2006).

Based on our RNA expression analysis, *Mbp* is downregulated in the CPu of 7 weeks old *Fmr1*-KO mice. Confirmation of these results by immunofluorescent labeling illustrated no changes in MBP protein levels in any of the analyzed brain regions. These results might suggest that although the mRNA MBP signal is downregulated, protein levels stay stable.

Maintaining physiological brain function requires both microglia and astrocytes (Reemst et al., 2016; Sloan and Barres, 2014; Sominsky et al., 2018). Several clinical studies suggest excessive microglial and astrocyte activation and inflammation in multiple brain regions of ASD individuals (Liao et al., 2020; Morgan et al., 2010; Suzuki et al., 2013; Vargas et al., 2005). In *Fmr1*-KO mice, activation of astrocytes in *Fmr1*-KO mice was already validated in cerebellar tissue of adult and in cortical tissue of young mice (Lee et al., 2019; Yuskaitis et al., 2010) and are confirmed to mediate the physiological neuronal development of these mice (Jacobs and Doering, 2010). Our study adds to these results as we found the overall signal for the astrocytic marker GFAP to be increased in the hippocampus of *Fmr*1-KO mice. In the hippocampus and in the cortex, the size of the GFAP-positive objects was increased, suggesting that astrocytes were activated in *Fmr1*-KO compared to C57BL/6J mice, as the cell size indicates the activation status of astrocytes.

We further observed microgliosis in the CPu that was characterized by a strong increase in the number of Iba1-positive objects and thus suggesting an increased number of microglia cells. Although our results confirm clinical study results, another study in *Fmr1*-KO mice did not detect microglial activation (Pacey et al., 2015; Yuskaitis et al., 2010). Lee and colleagues even reported a decrease in the number of Iba1-positive cells in the neocortex of young *Fmr1*-KO compared to wild type mice leading to a reduced synapse elimination (Lee et al., 2019). Further research on activation of astrocytes and microglia in *Fmr1*-KO mice is needed to elucidate its function for FXS.

In our gene expression analysis of CPu tissue from *Fmr1*-KO mice, we primarily focused on the two most upregulated genes, *Ndn* and *Ide*, while not fully exploring the strongest downregulated gene, *Gstm1*, which encodes glutathione S-transferase Mu1. As polymorphisms in this gene seem to positively influence some typical ASD symptoms, such as non-verbal communication and cognitive functioning (Mandic-Maravic et al., 2021), detailed research on *Gstm1* function in *Fmr1*-KO mice would clarify its role for FXS.

The pathway-level analysis by comprehensive functional enrichment analyses provides a system-level view of the transcriptomic consequences of *Fmr1* deficiency, reinforcing and extending the gene-level observations and offering potential mechanistic insights into the molecular underpinnings of FXS.

### 4.1 Limitations

Although this study reveals distinct changes in gene and protein expression within the CPu of *Fmr1*-KO mice, molecular differences cannot yet be directly linked to specific behavioral outcomes or broader disease mechanisms of this preclinical FXS model. Present results therefore represent an initial step toward identifying preclinical biomarkers rather than providing definitive evidence of causal pathways or therapeutic targets. Further studies are required to determine whether observed alterations influence fragile X–related phenotypes or can be safely and effectively modulated *in vivo* or even translated to the clinic.

Using *Fmr1*-KO mice at the age of 7 weeks limits our findings to a very early stage of FXS and may thus not reflect the pathology observed in juvenile or adult *Fmr1*-KO mice. Evaluation of further developmental stages of *Fmr1*-KO mice is thus needed to analyze if dysregulation of Ndn and Ide represents an age-specific phenomenon.

Our finding that only a proportion of NDN+ cells are ChAT+ raises the question of what cell type the remaining NDN+ cells are. As this analysis was beyond the scope of this manuscript, this question should be addressed in future studies.

For this study, the CPu of the left hemisphere of each animal was used for RNA analyses/sequencing while the right hemisphere was used for histological evaluations. This approach enabled us to keep the number of needed animals low but does not account for potential functional asymmetries as already observed in the rat CPu and FXS patients (Hoeft et al., 2007; Rothman and Glick, 1976; Thiel and Schwarting, 2001). Comparison of the right and left CPu of *Fmr1*-KO mice by RNA sequencing will be needed to evaluate lateralization effects.

The sample size of our study falls at the edge of recommended sample sizes for RNA-sequencing studies and is thus a significant limiting factor. For a cut-off of 2-fold expression differences, as shown for Nde and Ide, Halasz and colleagues therefore recommend using a sample size of at least 6 mice to decrease the false positive rate to be below 50% (Halasz et al., 2025). We therefore acknowledge that the statistical power of the current study may not be fully sufficient to capture the complete range of biological variability inherent to brain tissue, particularly given the multiple downstream analyses performed, including qPCR, immunofluorescence across brain regions, and ELISA. Furthermore, while Ndn and Ide emerge as interesting candidate markers from our transcriptomic analysis, we wish to emphasize that the current findings should be interpreted as hypothesis-generating rather than definitive biomarker proposals. A formal validation of these candidates as biomarkers would require larger, appropriately powered cohorts. Future studies should therefore include larger sample sizes to improve statistical power, more comprehensively capture biological variability, and strengthen the generalizability of these findings, both within the context of FXS and potentially across broader autism spectrum disorder animal models and human studies. The present study is best understood as an exploratory starting point for investigating the *Fmr1*-KO mouse model and the expression patterns of Ndn and Ide in FXS, laying the groundwork for more extensive validation in future work.

## 5 Conclusion

Our results indicate that *Ndn* and *Ide* exhibit differential gene expressions in the CPu of *Fmr1*-KO mice. Results were confirmed by immunohistochemistry, and, in the case of *Ide*, enzymatic activity was confirmed by measuring lower levels of insulin in the plasma of *Fmr1*-KO mice. NDN and IDE protein may therefore serve as potential tissue biomarkers for preclinical FXS research. However, validation of shown results is required to determine whether expression levels of NDN and IDE can be modulated by treatments that influence FMRP levels and consequently FXS symptoms.

## Data Availability

The original contributions presented in the study are publicly available. This data can be found here: https://fig share.com/s/6a093950c422f8293651 with the Doi: 10.6084/m9.figshare.33861790.
