# Supplementary material for: Gene expression analysis of Fmr1-KO mouse caudate putamen reveals new preclinical biomarkers for fragile X syndrome
Source: Front Neurosci. 2026 Oct 23;20:1833413. doi: 10.3389/fnins.2026.1833413 (PMC13741217; doi:10.3389/fnins.2026.1833413)
Supplement: Supplementary file 1 [file Data_Sheet_1.pdf]

Supplementary information

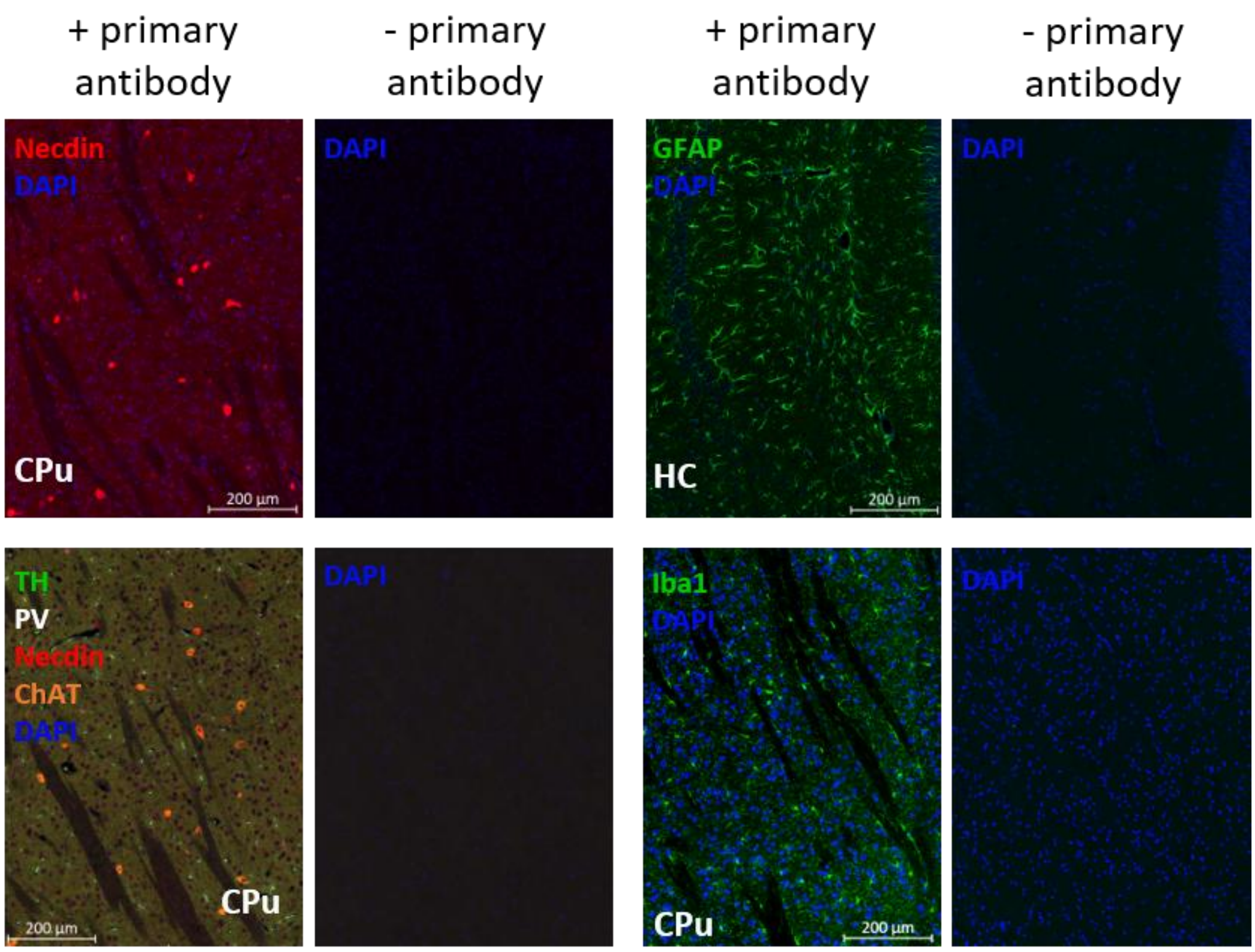

Suppl. Figure 1: Negative controls of immunofluorescent labeling. Representative images of 4 different labeling including (left) and omitting (right) the primary antibody. Scale bar: 200 µm for all images.

Suppl. Table 1: Numerical data of cell counts of Necdin and ChAT of co-expression analysis presented in Figure 3.

Group	Cell population	CPu	Hippocampus	Brainstem	Total
A	Necdin	579	667	35205	36451
	ChAT	381	2	1490	1873
	ChAT on Necdin	181	0	1173	1354
	Necdin on ChAT	192	1	1272	1465
B	Necdin	1167	1177	41408	43752
	ChAT	310	2	1755	2067
	ChAT on Necdin	278	2	1456	1736
	Necdin on ChAT	308	2	1595	1905

Suppl. Table 2: Correction of p-values for multiple comparison using Benjamini Hochberg false discovery rate of Necdin immunofluorescence analysis; data presented in Figure 1 B-C and in Figure 2 A-D. BS, brainstem; CPu, caudate putamen; HC, hippocampus.

region	area	p value	p corr	
CPU	IR area	0.001	0.004	**
CPU	obj dens	0.050	0.100	n.s.
CPU	obj int	0.340	0.371	n.s.
CPU	obj size	0.001	0.006	**
HC	IR area	0.010	0.030	*
HC	obj dens	0.810	0.810	n.s.
HC	obj int	0.050	0.120	n.s.
HC	obj size	0.001	0.012	*
BS	IR area	0.070	0.105	n.s.
BS	obj dens	0.230	0.276	n.s.
BS	obj int	0.110	0.147	n.s.
BS	obj size	0.007	0.012	*

Suppl. Table 3: Correction of p-values for multiple comparison using Benjamini Hochberg false discovery rate of co-expression analysis; data presented in Figure 3. BS, brainstem; CPu, caudate putamen; HC, hippocampus.

**Readout**	**area**	**p value**	**p corr**	
ChAT on Necdin	BS	0.644	0.644	n.s
	CPU	0.008	0.025	*
	HC	0.190	0.285	n.s
Necdin on ChAT	BS	0.410	0.492	n.s
	CPU	0.001	0.006	**
	HC	0.100	0.200	n.s

Suppl. Table 4: Correction of p-values for multiple comparison using Benjamini Hochberg false discovery rate of PV immunofluorescence analysis; data presented in Figure 4. CPu, caudate putamen; CX, cortex; HC, hippocampus.

**region**	**area**	**p value**	**p corr**	
CX	IR area	0.020	0.240	n.s
CX	obj dens	0.050	0.300	n.s
CX	obj int	0.280	0.560	n.s
CX	obj size	0.300	0.514	n.s
CPU	IR area	0.880	0.880	n.s
CPU	obj dens	0.840	0.916	n.s
CPU	obj int	0.580	0.870	n.s
CPU	obj size	0.650	0.780	n.s
HC	IR area	0.200	0.480	n.s
HC	obj dens	0.600	0.800	n.s
HC	obj int	0.090	0.360	n.s
HC	obj size	0.150	0.450	n.s

Suppl. Table 5: Correction of p-values for multiple comparison using Benjamini Hochberg false discovery rate of MBP immunofluorescence analysis; data presented in Figure 6. CC, corpus callosum; CPu, caudate putamen; CX, cortex; HC, hippocampus.

region	area	p value	p corr	
CPU	IR area	0.970	1.035	n.s
CPU	obj dens	0.040	0.128	n.s
CPU	obj int	0.190	0.434	n.s
CPU	obj size	0.030	0.120	n.s
CX	IR area	0.560	0.815	n.s
CX	obj dens	0.030	0.240	n.s
CX	obj int	0.990	0.990	n.s
CX	obj size	0.600	0.800	n.s
HC	IR area	0.740	0.911	n.s
HC	obj dens	0.070	0.187	n.s
HC	obj int	0.900	1.029	n.s
HC	obj size	0.200	0.400	n.s
CC	IR area	0.030	0.160	n.s
CC	obj dens	0.350	0.560	n.s
CC	obj int	0.210	0.373	n.s
CC	obj size	0.008	0.128	n.s

Suppl. Table 6: Correction of p-values for multiple comparison using Benjamini Hochberg false discovery rate of GFAP immunofluorescence analysis; data presented in Suppl Figure 3. CPu, caudate putamen; CX, cortex; HC, hippocampus.

region	area	p value	p corr	
CPU	IR area	0.03	0.045	*
CPU	obj size	0.61	0.610	n.s
CX	IR area	0.3	0.360	n.s
CX	obj size	0.004	0.012	*
HC	IR area	0.009	0.018	*
HC	obj size	0.001	0.006	**

Suppl. Table 7: Correction of p-values for multiple comparison using Benjamini Hochberg false discovery rate of Iba1 immunofluorescence analysis; data presented in Suppl Figure 4. CPu, caudate putamen; CX, cortex; HC, hippocampus.

region	area	p value	p corr	
CX	IR area	0.06	0.120	n.s
CX	obj density	0.06	0.090	n.s
CPU	IR area	0.001	0.006	**
CPU	obj density	0.001	0.003	**
HC	IR area	0.32	0.320	n.s
HC	obj density	0.22	0.264	n.s

Suppl. Table 8: Full list of up- and downregulated genes in the caudate putamen of *Fmr1*-KO mice compared to C57BL/6J mice based on RNA sequencing.

Name	Upregulated Genes	baseMean	log2FoldChange	p-value	adjusted p-value
NDN	necdin [Source:MGI Symbol;Acc:MGI:97290]	1167,863921	-0,848923074	2,69E-25	1,73E-21
Ide	insulin degrading enzyme [Source:MGI Symbol;Acc:MGI:96412]	188,2709075	-0,696718342	1,58E-15	6,79E-12
FGFBP3	fibroblast growth factor binding protein 3 [Source:MGI Symbol;Acc:MGI:1919764]	48,9739596	-0,442476121	6,39E-06	0,008069641
RAB39B	RAB39B, member RAS oncogene family [Source:MGI Symbol;Acc:MGI:1915040]	209,3702848	-0,438811634	1,21E-06	0,001954054
CAR10	carbonic anhydrase 10 [Source:MGI Symbol;Acc:MGI:1919855]	80,06927851	-0,366165229	0,000308255	0,084461855
Snrpc	U1 small nuclear ribonucleoprotein C [Source:MGI Symbol;Acc:MGI:109489]	82,31066765	-0,366027058	0,000204744	0,064309718
FCRLS	Fc receptor-like S, scavenger receptor [Source:MGI Symbol;Acc:MGI:1933397]	113,0845326	-0,359659357	0,000231047	0,069195763
SST	somatostatin [Source:MGI Symbol;Acc:MGI:98326]	2088,257479	-0,354783319	3,34E-07	0,000859597
ETV5	ets variant 5 [Source:MGI Symbol;Acc:MGI:1096867]	243,5799019	-0,310393185	0,000255331	0,074730642
AQP4	aquaporin 4 [Source:MGI Symbol;Acc:MGI:107387]	377,1669718	-0,255889066	0,000324598	0,087086816
NPY	neuropeptide Y [Source:MGI Symbol;Acc:MGI:97374]	782,7784711	-0,235477455	0,000195666	0,062994739
YWHAG	tyrosine 3-monooxygenase/tryptophan 5-monooxygenase activation protein, gamma polypeptide [Source:MGI Symbol;Acc:MGI:108109]	2564,438892	-0,219508118	3,05E-05	0,019996653
BEX2	brain expressed X-linked 2 [Source:MGI Symbol;Acc:MGI:1338017]	1490,611134	-0,201563715	0,000193968	0,062994739
**Name**	**Downregulated Genes**	**baseMean**	**log2FoldChange**	**p-value**	**adjusted p-value**
MBP	myelin basic protein [Source:MGI Symbol;Acc:MGI:96925]	41630,91741	0,187111291	0,000105594	0,049887016
CNP	2',3'-cyclic nucleotide 3' phosphodiesterase [Source:MGI Symbol;Acc:MGI:88437]	4005,936046	0,203189556	0,000122935	0,053122949
RGS4	regulator of G-protein signaling 4 [Source:MGI Symbol;Acc:MGI:108409]	8919,9955	0,217290428	0,000262296	0,075063421
PLP1	proteolipid protein (myelin) 1 [Source:MGI Symbol;Acc:MGI:97623]	58438,67856	0,217621331	2,08E-05	0,018389979
SLC44A1	solute carrier family 44, member 1 [Source:MGI Symbol;Acc:MGI:2140592]	564,1979977	0,238606184	0,000138386	0,054003933
POU3F1	POU domain, class 3, transcription factor 1 [Source:MGI Symbol;Acc:MGI:101896]	902,8405986	0,253306821	0,000376902	0,097148191
FTH1	ferritin heavy polypeptide 1 [Source:MGI Symbol;Acc:MGI:95588]	20624,33561	0,256452037	3,15E-05	0,019996653
MAL	myelin and lymphocyte protein, T cell differentiation protein [Source:MGI Symbol;Acc:MGI:892970]	4680,134952	0,272501704	4,09E-05	0,02391747
NDUFA1	NADH:ubiquinone oxidoreductase subunit A1 [Source:MGI Symbol;Acc:MGI:1929511]	544,1176759	0,274006544	0,000285485	0,079923267
UNC13C	unc-13 homolog C [Source:MGI Symbol;Acc:MGI:2149021]	1789,195969	0,275960973	3,26E-05	0,019996653
UBE2M	ubiquitin-conjugating enzyme E2M [Source:MGI Symbol;Acc:MGI:108278]	389,1015943	0,294384879	0,000149707	0,056703755
ABHD4	abhydrolase domain containing 4 [Source:MGI Symbol;Acc:MGI:1915938]	380,9407227	0,302168352	7,14E-05	0,038299829
PRXL2B	peroxiredoxin like 2B [Source:MGI Symbol;Acc:MGI:1913719]	427,0152601	0,306035701	0,000377187	0,097148191
TTC39B	tetratricopeptide repeat domain 39B [Source:MGI Symbol;Acc:MGI:1917113]	283,2833935	0,317764995	2,43E-05	0,018389979
GRB14	growth factor receptor bound protein 14 [Source:MGI Symbol;Acc:MGI:1355324]	368,2495357	0,31804737	0,000165105	0,059067576
BTBD1	BTB (POZ) domain containing 1 [Source:MGI Symbol;Acc:MGI:1933765]	239,2337125	0,325388725	4,93E-05	0,027610565
GRHPR	glyoxylate reductase/hydroxypyruvate reductase [Source:MGI Symbol;Acc:MGI:1923488]	153,7406424	0,333052693	0,00023055	0,069195763
PIPOX	pipecolic acid oxidase [Source:MGI Symbol;Acc:MGI:1197006]	18,89550322	0,34583035	0,00013443	0,054003933
PTGDS	prostaglandin D2 synthase (brain) [Source:MGI Symbol;Acc:MGI:99261]	4765,046684	0,35344786	1,83E-05	0,018084471
PDZD2	PDZ domain containing 2 [Source:MGI Symbol;Acc:MGI:1922394]	277,2988055	0,362381274	2,16E-05	0,018389979
TNS1	tensin 1 [Source:MGI Symbol;Acc:MGI:104552]	277,4370081	0,365099256	2,52E-06	0,003600566
TPPP3	tubulin polymerization-promoting protein family member 3 [Source:MGI Symbol;Acc:MGI:1915221]	378,2568363	0,372013796	0,000123753	0,053122949
ANO2	anoctamin 2 [Source:MGI Symbol;Acc:MGI:2387214]	59,05883602	0,377657914	0,000190317	0,062994739
AMZ1	archaelysin family metallopeptidase 1 [Source:MGI Symbol;Acc:MGI:2442258]	68,27325318	0,377958578	0,000184073	0,062994739
CD59A	CD59a antigen [Source:MGI Symbol;Acc:MGI:109177]	324,2593211	0,378568372	1,67E-05	0,017923007
RARRES2	retinoic acid receptor responder (tazarotene induced) 2 [Source:MGI Symbol;Acc:MGI:1918910]	57,21689375	0,381361154	0,000165121	0,059067576
CRYZL2	crystallin zeta like 2 [Source:MGI Symbol;Acc:MGI:2448516]	29,18549456	0,382930453	9,80E-05	0,048550882
MAFA	v-maf musculoaponeurotic fibrosarcoma oncogene family, protein A (avian) [Source:MGI Symbol;Acc:MGI:2673307]	59,74551124	0,388551805	0,000128997	0,053587783
LYZ2	lysozyme 2 [Source:MGI Symbol;Acc:MGI:96897]	55,10955357	0,390357691	0,000108467	0,049887016
ABI3BP	ABI gene family, member 3 (NESH) binding protein [Source:MGI Symbol;Acc:MGI:2444583]	34,43585726	0,396171085	7,78E-05	0,040078171
ARHGDIB	Rho, GDP dissociation inhibitor (GDI) beta [Source:MGI Symbol;Acc:MGI:101940]	137,8413159	0,397483675	3,04E-05	0,019996653
PITPNM3	PITPNM family member 3 [Source:MGI Symbol;Acc:MGI:2685726]	108,3441702	0,426113101	6,89E-06	0,008069641
GM45441	predicted gene 45441 [Source:MGI Symbol;Acc:MGI:5791277]	39,42708405	0,428271921	2,37E-05	0,018389979
APOD	apolipoprotein D [Source:MGI Symbol;Acc:MGI:88056]	2795,950812	0,43809561	5,48E-11	1,76E-07
RIDA	reactive intermediate imine deaminase A homolog [Source:MGI Symbol;Acc:MGI:1095401]	389,954328	0,449206288	6,89E-07	0,00126695
GSTM1	glutathione S-transferase, mu 1 [Source:MGI Symbol;Acc:MGI:95860]	1452,089528	0,501536341	4,75E-07	0,00101974
FMR1	fragile X mental retardation 1 [Source:MGI Symbol;Acc:MGI:95564]	126,1756505	1,134210764	3,15E-34	4,06E-30

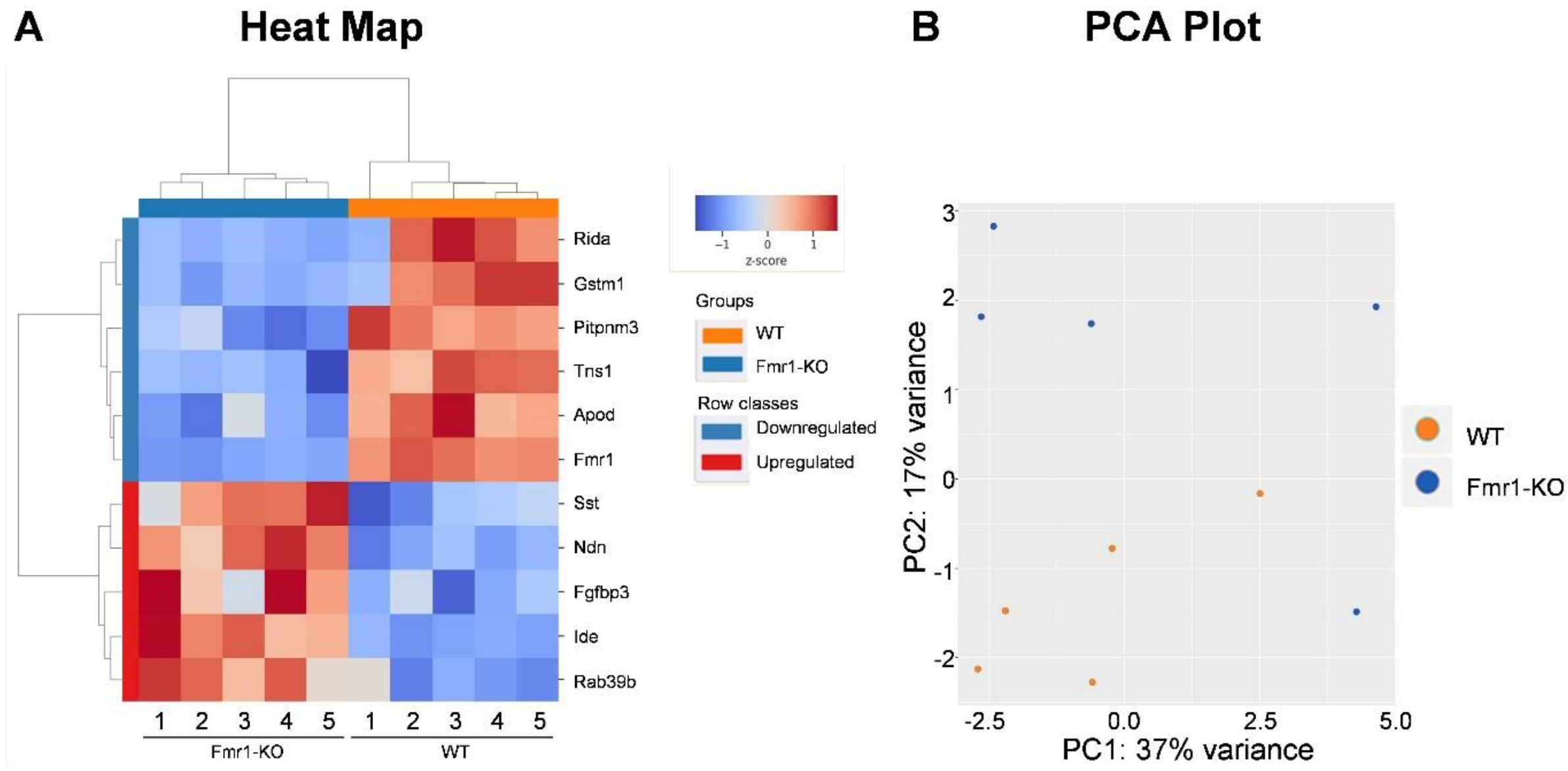

Suppl. Figure 2: Heat map (**A**) and principle component analysis plot **(B)** of up- and downregulated genes in the caudate putamen of *Fmr1*-KO mice compared to C57BL/6J mice based on RNA sequencing.

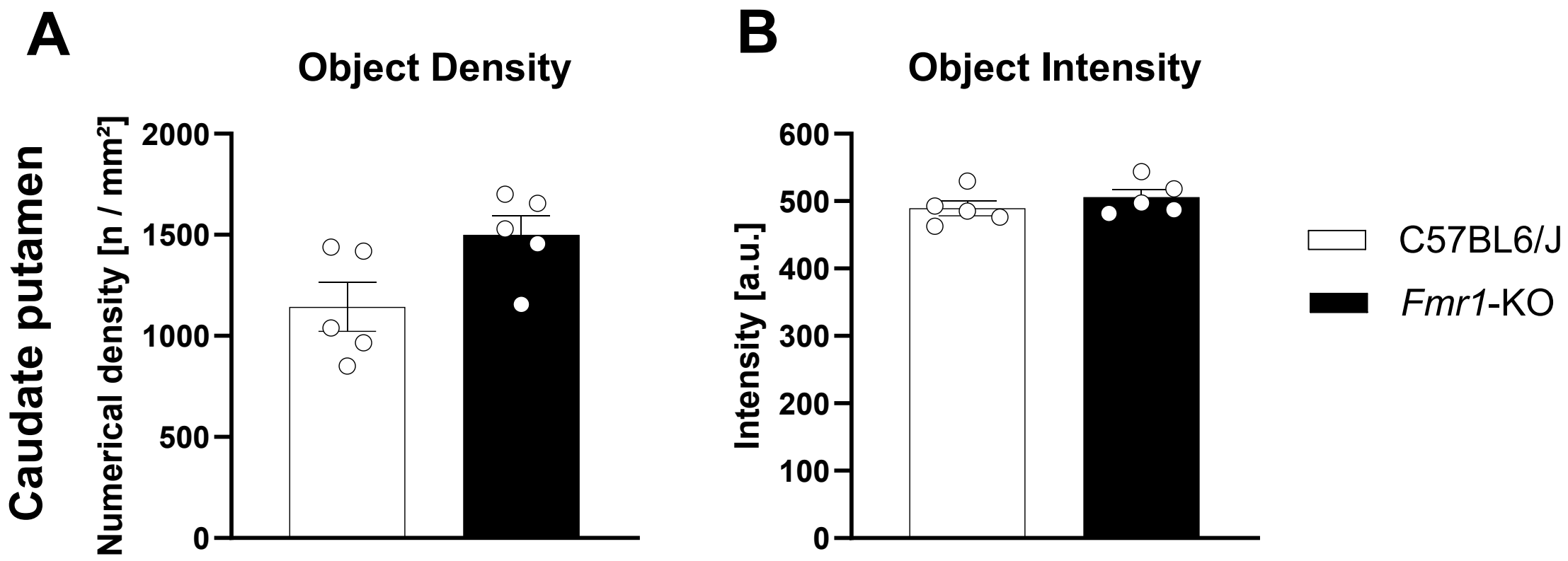

Suppl. Figure 3: Quantification of Necdin protein expression in the caudate putamen of *Fmr1*-KO and C57BL/6J mice. A: Necdin (NDN) object density in n/mm^2 and **B**: necdin object intensity in the caudate putamen. **A and B**: n= 5 per group; mean ± SEM; unpaired t-test corrected for multiple comparisons using Benjamini Hochberg false discovery rate.

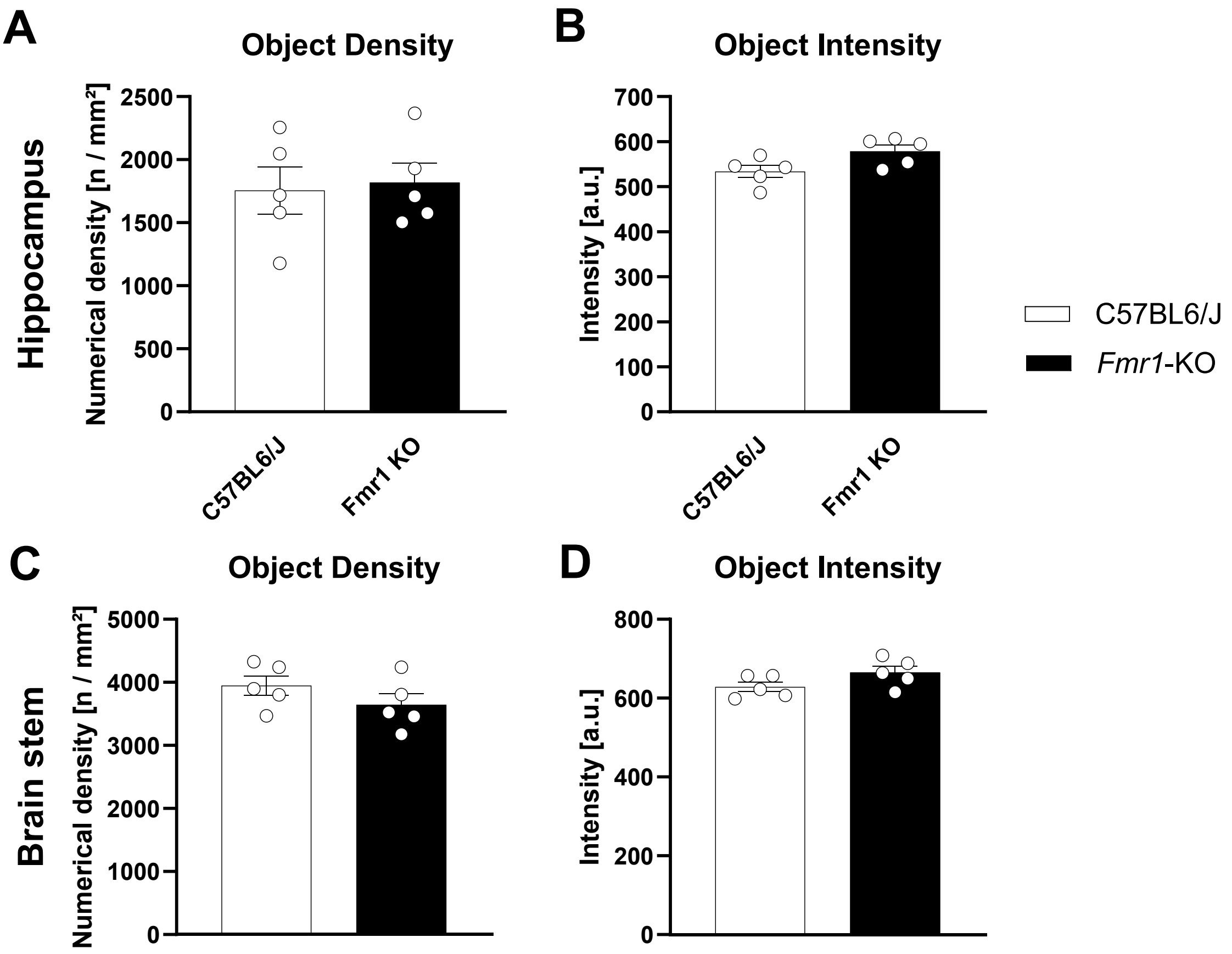

Suppl. Figure 4: Quantification of Necdin protein expression in the hippocampus (A, B) and brainstem (C,D) of *Fmr1*-KO and C57BL/6J mice. A, C: Necdin (NDN) object density in n/mm^2 and **B, D**: necdin object intensity in the caudate putamen. **A-D**: n= 5 per group; mean ± SEM; unpaired t-test corrected for multiple comparisons using Benjamini Hochberg false discovery rate.

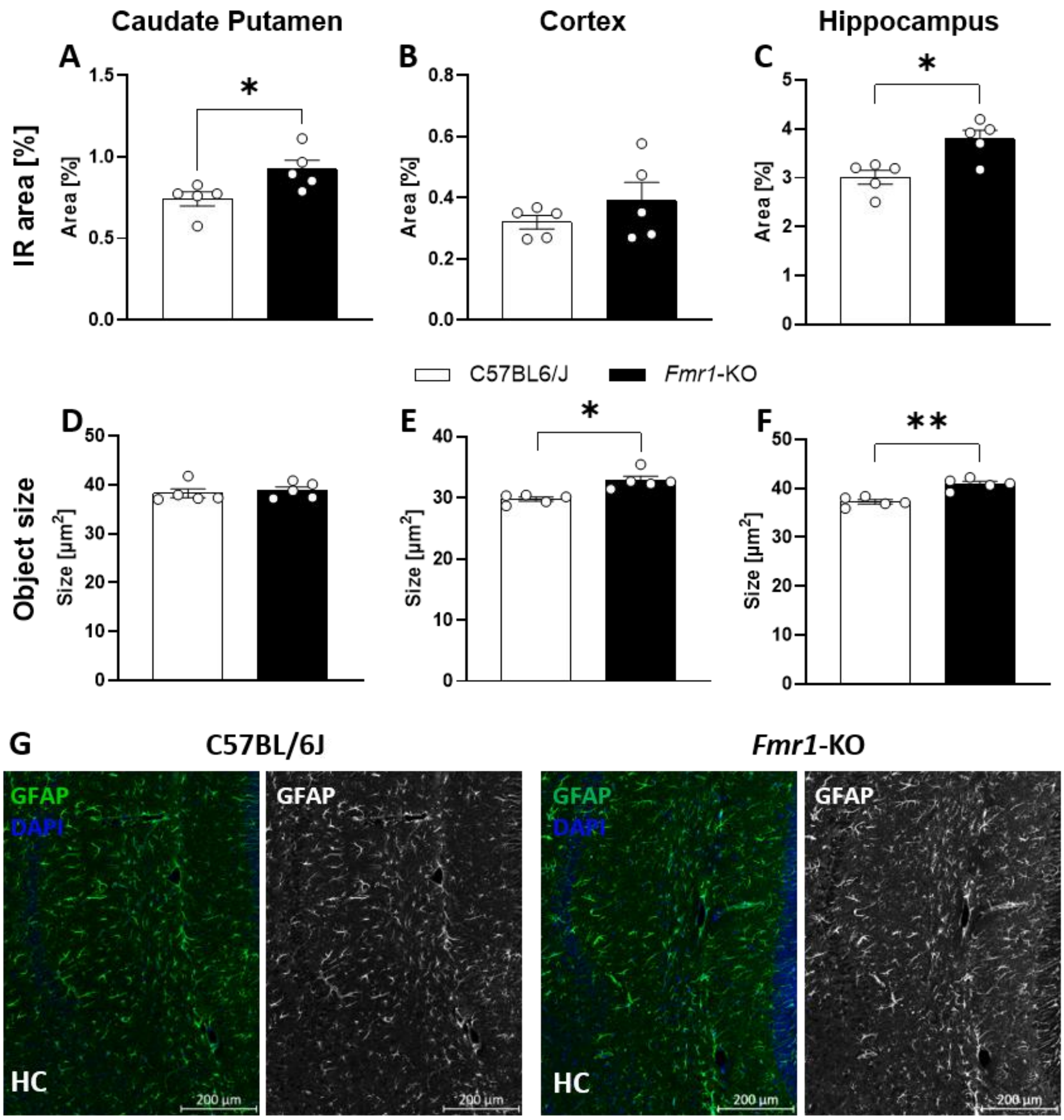

Suppl. Figure 5: Quantification of the astrocytic marker GFAP in different brain regions of *Fmr1*-KO and C57BL/6J mice. GFAP immunoreactive (IR) area in percent **(A-C)** and GFAP object size in µm^2 **(D-F)** in the caudate putamen **(A, D)**, cortex **(B, E)**, and hippocampus **(C, F)**. **A-F**: n= 5 per group; mean ± SEM; unpaired *t*-test corrected for multiple comparisons using Benjamini Hochberg false discovery rate; *p < 0.05, **p < 0.01. **G**: Representative images of GFAP (green) in the hippocampus (HC) of C57BL/6J and *Fmr1*-KO mice. DAPI (blue) was used to counterstain nuclei. DAPI, 4',6-diamidin-2-phenylindole.

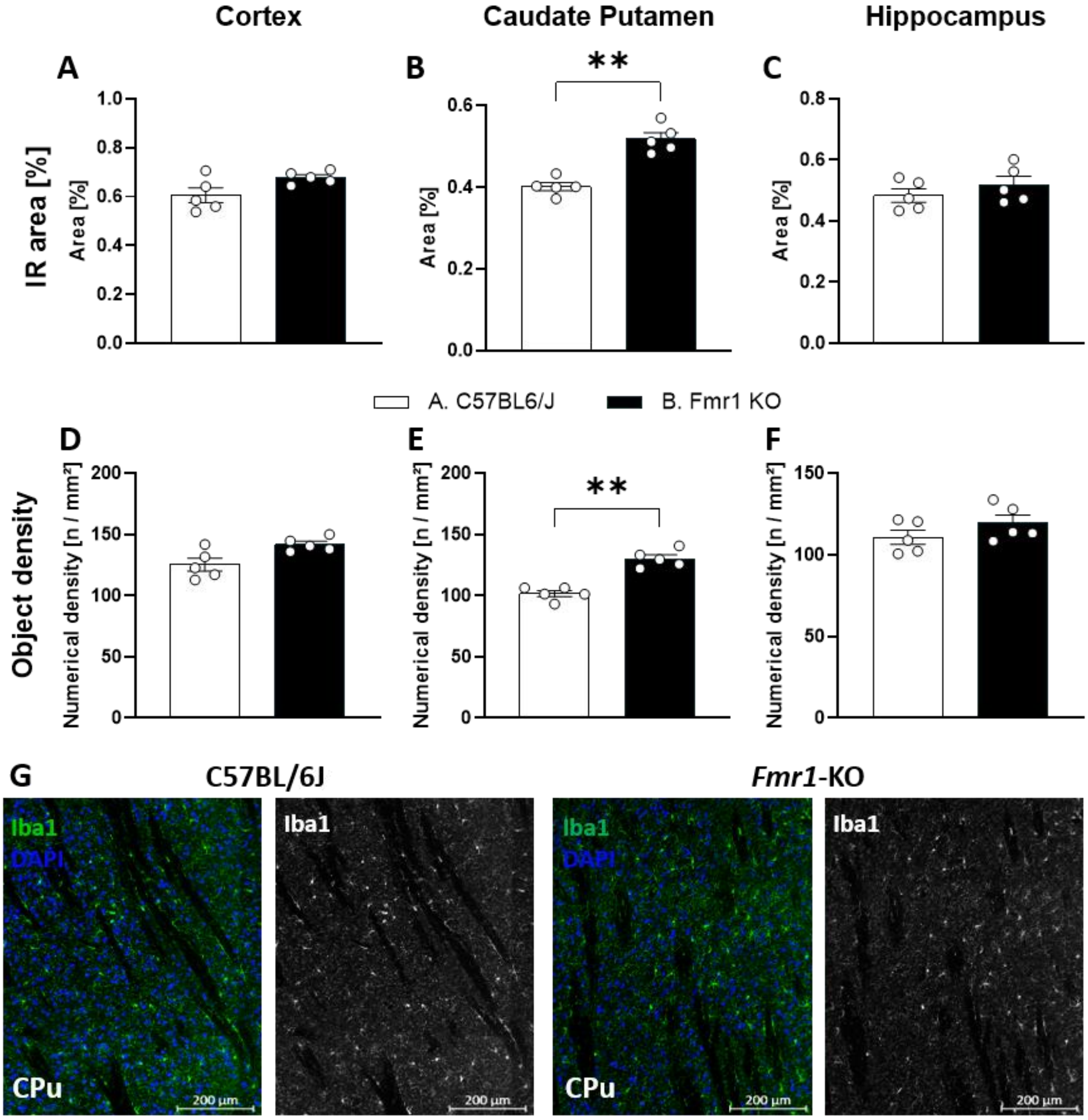

Suppl. Figure 6: Quantification of the microglial marker Iba1 in different brain regions of *Fmr1*-KO and C57BL/6J mice. Iba1 immunoreactive (IR) area in percent **(A-C)** and Iba1 object density in n/mm2 **(D-F)** in the caudate putamen (**A, D**), cortex (**B, E**), and hippocampus (**C, F**). **A-F**: n= 5 per group; mean ± SEM; unpaired *t*-test corrected for multiple comparisons using Benjamini Hochberg false discovery rate; **p < 0.01. **G**: Representative images of Iba1 (green) in the caudate putamen (CPu) of C57BL/6J and *Fmr1*-KO mice. NeuN (blue) was used to counterstain neuronal nuclei.
